# Anisotropic Thermal Conductivity of Inkjet-Printed 2D Crystal Films: Role of the Microstructure and Interfaces

**DOI:** 10.3390/nano12213861

**Published:** 2022-11-01

**Authors:** Mizanur Rahman, Khaled Parvez, Giorgia Fugallo, Chaochao Dun, Oliver Read, Adriana Alieva, Jeffrey J. Urban, Michele Lazzeri, Cinzia Casiraghi, Simone Pisana

**Affiliations:** 1 Department of Physics and Astronomy, York University, Toronto, ON M3J 1P3, Canada; 2 School of Chemistry, Manchester University, Manchester M13 9PL, UK; 3 Laboratoire de Thermique et Energie de Nantes, CNRS UMR 6607, Université de Nantes, 44306 Nantes, France; 4 Lawrence Berkeley National Laboratory, Berkeley, CA 94720, USA; 5 Institut de Minéralogie, de Physique des Matériaux et de Cosmochimie, CNRS UMR 7590, Sorbonne Université, 75005 Paris, France; 6 Department of Electrical Engineering and Computer Science, York University, Toronto, ON M3J 1P3, Canada

**Keywords:** thermal conductivity, 2D materials, ink-jet printing, density functional theory

## Abstract

Two-dimensional (2D) materials are uniquely suited for highly anisotropic thermal transport, which is important in thermoelectrics, thermal barrier coatings, and heat spreaders. Solution-processed 2D materials are attractive for simple, low-cost, and large-scale fabrication of devices on, virtually, any substrate. However, to date, there are only few reports with contrasting results on the thermal conductivity of graphene films, while thermal transport has been hardly measured for other types of solution-processed 2D material films. In this work, inkjet-printed graphene, h-BN and MoS${}_{2}$ films are demonstrated with thermal conductivities of ∼10 Wm${}^{-1}$K${}^{-1}$ and ∼0.3 Wm${}^{-1}$K${}^{-1}$ along and across the basal plane, respectively, giving rise to an anisotropy of ∼30, hardly dependent on the material type and annealing treatment. First-principles calculations indicate that portion of the phonon spectrum is cut-off by the quality of the thermal contact for transport along the plane, yet the ultra-low conductivity across the plane is associated with high-transmissivity interfaces. These findings can drive the design of highly anisotropic 2D material films for heat management applications.

## 1. Introduction

Thermal conductivity is one of the most important properties of a material, while the range of thermal conductivity values spanned by fully dense materials is limited to within only 4 orders of magnitude [1], accessing these limits is crucially important for heat management in broad applications areas such as computing [2], energy generation [3,4] and storage [5], and space exploration [6]. In some applications, such as thermoelectric generators [7], electronic packaging [8] and data storage [9], it is highly desirable to have a high thermal conductivity along one direction, typically in the plane of the substrate ($K_{\|}$), and a low thermal conductivity in the orthogonal direction, out of the plane of the substrate ($K_{⊥}$). To this end, combining these requirements would mean having insulating or semiconducting materials with light atoms, strong bonds, low anharmonicity and large crystal size in-plane [10], while having large mass contrast, weak bonds and lack of long-range order out-of-plane [11,12].

2D materials, their layered heterostructures or intercalated compounds can satisfy these requirements [13,14,15]. The body of knowledge on heat transport of layered materials has provided some insights into structure-property relationships, but less is known about the ultrahigh/ultralow conductivity limiting cases or about how $K_{\|}$ and $K_{⊥}$ may be related. Achieving high $K_{\|}$ values leads to selecting crystals such as graphite and h-BN, and the conductivity is then limited by crystal size [16]. However, once these materials are selected, it is not clear how to significantly lower $K_{⊥}$ much below the bulk value, since the differences in phonon dispersions along and across the basal plane sets intrinsic limits to the heat transport. One approach to lowering $K_{⊥}$ involves heterogeneously layered crystal structures that are composed of alternating 2D crystals of different composition [12,14,17], but these are difficult and expensive to fabricate in large quantities. Similarly, fabricating thin film systems with high interface densities can also decrease transport across the plane [18,19], but suffer similar drawbacks. Another approach is to limit the out of plane crystal size, thereby introducing additional boundary scattering [16,20]. Using thin films below 10 nm, however, limits the use of thin crystals for thermal applications. Graphite and h-BN laminates composed of micron-sized flakes a few atomic layers thick could achieve overall arbitrary thickness while maintaining low $K_{⊥}$, and these have been shown to posses high $K_{\|}$[21,22], but the only studies available on anisotropy and $K_{⊥}$ deal with films having relatively porous structures made by evaporation or vacuum filtration (see for example Refs. [22,23,24]), and therefore $K_{⊥}$ depends highly on fabrication conditions and amount of compression the films are subjected to.

Scalable and inexpensive fabrication approaches such as solution-processing [25] present very attractive manufacturing routes to assemble high-quality 2D crystal laminates. However, while heat transport has been extensively studied for single crystals and crystalline thin films [12,14,15,16,17,21,22], only very few works have provided a detailed characterization of both $K_{\|}$ and $K_{⊥}$ in films of solution-processed 2D materials (a table of the state of art is provided in Table 1). In particular, in the case of pristine graphene, only five studies have been reported [23,24,26,27,28], showing anisotropy value $A_{Kth}=K_{\|}/K_{⊥}$ in the range 70–675, where the highest values are typically obtained by high-temperature annealing at 1000 ${}^{{}^{\circ}}$C [23]. Furthermore, only two works report the thermal conductivities for other 2D material [20,29]. In addition all the studies were performed on thick laminates (thickness above 1 μm), mostly produced by vacuum filtration, which is known to give a poor control on the assembly of the flakes, compared to other techniques, such as ink-jet printing [30]. Unfortunately, the results from the previous works are difficult to compared due to the different materials properties and processing conditions used. Because of that, a full understanding on thermal conductivity of solution-processed 2D crystal films and how it relates to their microstructure and quality of the interfaces is still lacking. In particular, no work reported to date for these materials includes theoretical models that consider non-diffusive heat transport, which is necessary when the structure size is comparable to the heat carrier mean free path.

This work provides a comprehensive study on the thermal transport in printed films by looking at different 2D materials, different flake sizes, film thickness and post-processing conditions. The films studied in this work are composed of a dense and aligned stack of few-layer 2D crystals, and are made by inkjet printing, a cheap and scalable technique, without the use of high temperature annealing or harsh post-processing. We demonstrate that inkjet-printed films made of defect-free graphene, h-BN and MoS${}_{2}$ nanosheets yield ultra-low $K_{⊥}$, i.e., well below the respective bulk phase and lower than the thermal conductivity of amorphous SiO_2_ (glass). $A_{Kth}$ is found to be ∼30, independent of the chemical composition of the 2D crystal and the films thickness (<400 nm). Ab-initio modeling shows that even for such low $K_{⊥}$, energy transport is essentially ballistic across near-ideal interfaces. This is a remarkable result considering that previous reports, demonstrating comparable thermal conductivities, are obtained for either the disordered amorphous limit [11,31] or by maximizing atomic mass contrast in layered compounds [12,14]. At the same time, the measured $K_{\|}$ of these films are found to be very similar to one another, while this may at first be surprising considering the nearly 2 orders of magnitude difference in intrinsic thermal conductivities of the constituent crystals, the weak flake bonding filters high-energy phonon modes and limits the phonon spectrum contributing to the overall $K_{\|}$. This highlights fundamental differences from previous studies on single crystal 2D materials.

## 2. 2D Crystal Film Preparation and Characterization

Graphene, h-BN, and MoS${}_{2}$ inks were prepared by stabilizer-assisted liquid phase exfoliation, as previously reported [25] (further details in the Supplementary Information). Two graphene dispersions have been prepared containing nanosheets with average lateral size of 170 nm and 90 nm, while h-BN and MoS${}_{2}$ dispersions contain nanosheets with average lateral size of 160 nm and 50 nm, respectively, as determined by atomic force microscopy (more details of ink preparation and nanosheet characterization in the Supporting Information). According to atomic force microscopy, the average flake thickness is 4.4 nm for MoS_2_ and 6.5 nm for graphene and h-BN, which however cannot be converted directly into number of layers, as the value also includes the presence of residual stabilizer on the surfaces of the nanosheets. A more precise estimate of the actual thickness was obtained by transmission electron microscopy, which indicates 4–7 layers on average [32]. Taking a value of 6 layers, this corresponds to average thickness of 2 nm for h-BN and the two graphene dispersions, and 4 nm for MoS${}_{2}$, and this is the flake thickness used in the theoretical analysis.

**Figure 1:**
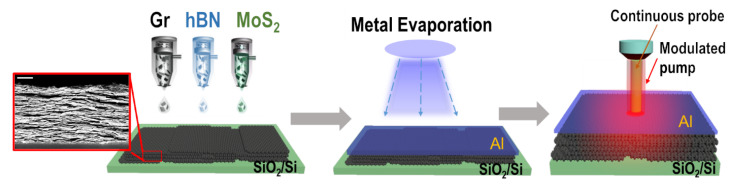
Schematic of the sample preparation for thermal conductivity measurements by pump-probe frequency-domain thermoreflectance. The 2D-material based ink is first prepared by assisted-liquid phase exfoliation, and then inkjet printed on silicon substrate and coated with an Al metal layer. The films are characterized by a dense array of 2D crystal nanosheets (see inset, showing the film cross section, taken by scanning electron microscopy; scale bar = 1 μm).

**Figure 2:**
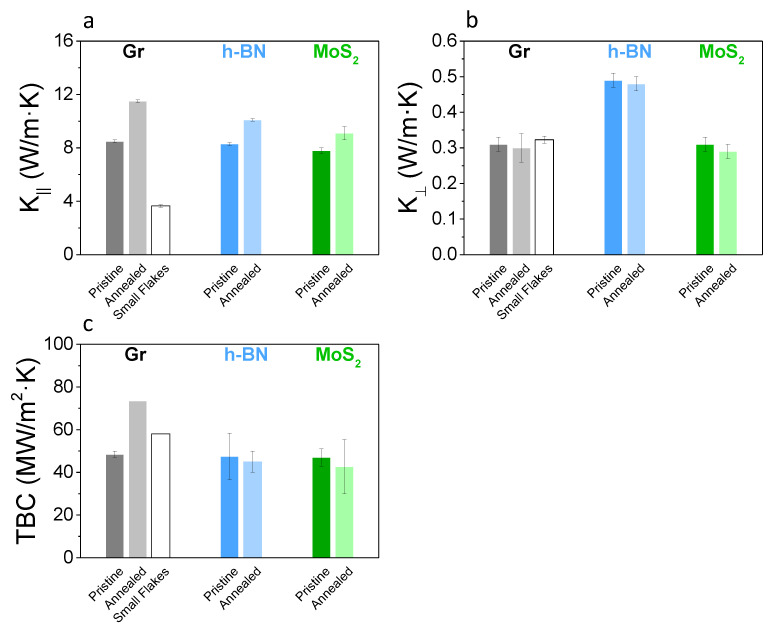
Thermal conductivity and thermal boundary conductance (TBC) of inkjet-printed 2D crystal films. Each value is the average of the measurements obtained for films of varying thickness, where each film thickness is measured on several different locations. As-deposited films before annealing are labeled as “pristine”, otherwise films were annealed in air at 150 ${}^{{}^{\circ}}$C. Smaller diameter flakes obtained through a longer sonication treatment of the ink solution (no annealing) are labeled as “small flakes”. The in-plane thermal conductivity (**a**) shows remarkable similarity for crystals having intrinsically very different bulk thermal conductivities. This quantity is affected mostly by flake size and quality of interface among flakes (see text and Figure 4). The out-of-plane thermal conductivities (**b**) are ultra-low, a repercussion of the small thickness of the flakes, but associated with high transmissivity interfaces (Figure 4). The TBC of the printed film with Al (**c**) shows values that are typical of metal interfaces with 2D materials.

**Figure 3:**
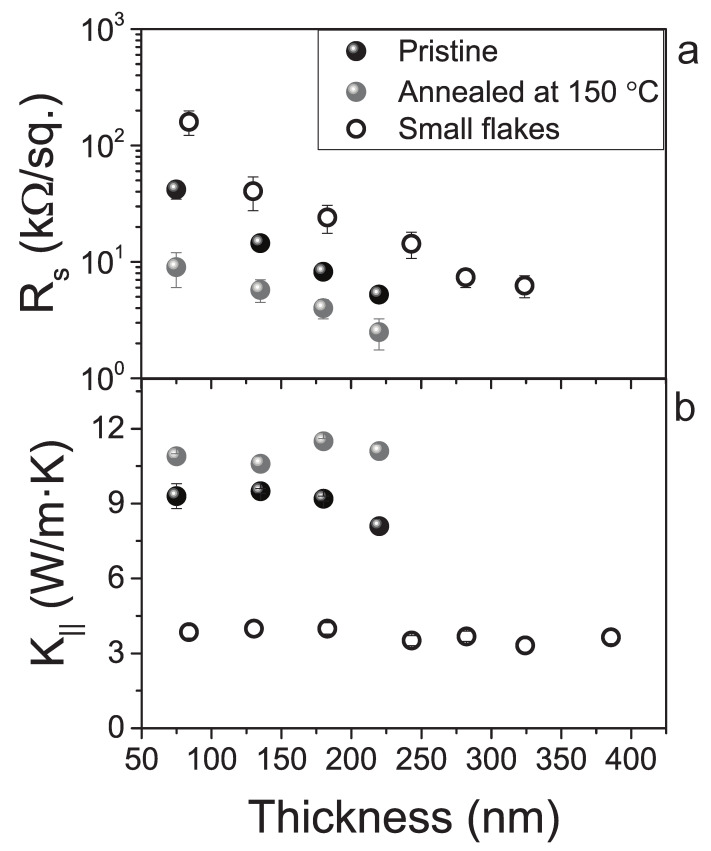
Thickness dependence of sheet resistance and in-plane thermal conductivity of inkjet-printed graphene films. The sheet resistance (**a**) shows a marked change with film thickness, size of the flakes and annealing. The in-plane thermal conductivity (**b**) shows negligible dependence on film thickness and annealing, whereas flake size has a more marked contribution. The thermal conductivity is expected to stay constant with film thickness if the microstructure is unaltered. Annealing increases the electrical conductivity more than the thermal conductivity. Flake size alters the boundary scattering length scale, as indicated in Figure 4.

The thickness of the inkjet-printed films, using the same ink, is in the range 50–400 nm by changing the number of printed passes. Some of the films were also annealed at 150 ${}^{{}^{\circ}}$C in air. The microstructure consists of a rather dense laminate of stacked flakes, Figure 1. Additional images of the cross sections of the pristine and annealed films can be found in the Supplementary Information, in addition to images of films obtained from the same ink by using vacuum filtration. We note that the microscopy image of the film microstructure is obtained for much thicker films than those used here, and the apparent presence of voids in the cross-section is likely the result of the film preparation prior to imaging. The upper bound for the amount of residual stabilizer in the films is between 3 and 10%. A thin layer of Al, ∼50 nm thick, is deposited on the surface of the film, as shown in Figure 1, to enable the measurement of its thermal properties through frequency domain thermoreflectance (FDTR) [33]. In this method, the phase lag between the heat flux generated by a sinusoidally modulated pump laser and the oscillating surface temperature observed by a reflected probe laser is measured as function of modulation frequency. The resulting frequency dependence of the thermal phase contains information about the thermal properties of the sample, and is used to obtain the values for $K_{\|}$, $K_{⊥}$ of the 2D crystal film and the thermal boundary conductance (TBC) between the 2D crystal film and the top Al layer (Figure 2). FDTR measurements were performed at room temperature as previously described [33,34]. Briefly, a pump laser operating at 515 nm is modulated from 50 kHz to 50 MHz and is focused using a 40× objective on the surface of the films coated with a 50 nm Al layer. The resulting changes in the surface temperature are detected by a 785 nm probe laser and are phase-shifted with respect to the pump modulation. The thermal phase as function of modulation frequency is then fit to a multilayered anisotropic solution of the diffusive heat equation to determine the unknown thermal parameters of interest. Further details of the thermal model, sensitivity to measured values and sources of error are provided in the Supplementary Information. FDTR measurements were performed in several locations in each printed film. Each measurement was fit to a multilayer diffusive model and errors for each fit were obtained through a Monte Carlo routine that propagates uncertainties in experimental and assumed parameters [34]. Results for each film thickness or material type are reported by taking the statistical average and standard deviation of the relevant ensemble, though the thermal properties for each material are reported for all film thickness values as these were found to be independent on thickness (Figure 3). Our results show that the values of the thermal boundary conductance, TBC, between Al and the different 2D crystal films are very similar, near 50 MWm${}^{-2}$K${}^{-1}$, Figure 2c. These values compare favorably with those reported for Al/graphite [35] and Al/MoS${}_{2}$ [36], though we are not aware of previous reports for the Al/h-BN interface. Generally, the TBC at metal-2D crystal interfaces is low compared to that of most metal-dielectric interfaces. Interfacial phonon mismatch, metal bond adhesion strength [35] and phonon focusing [37] affect the TBC in these systems.

## 3. Results

Figure 2a shows that the in-plane thermal conductivities of the films are low and remarkably similar. The $K_{\|}$ for the as-prepared films of graphene, h-BN and MoS${}_{2}$ are all within a few percent of 8 Wm${}^{-1}$K${}^{-1}$. This is at first surprising, considering that the intrinsic values of the thermal conductivities along the basal planes span a large range: ∼2000 Wm${}^{-1}$K${}^{-1}$ for graphite [40], ∼400 Wm${}^{-1}$K${}^{-1}$ for h-BN [41] and ∼100 Wm${}^{-1}$K${}^{-1}$ for MoS${}_{2}$[42]. As we shall discuss later, the similarity is largely coincidental, but has a common microscopic origin, as the $K_{\|}$ is dominated by the allowed phonon modes that transmit at the interface of overlapping flakes. This is brought into evidence by comparing the results for graphene films made by dispersions containing nanosheets with different average size: the decrease in size from 200 nm to 80 nm reduces $K_{\|}$ from 8.5 Wm${}^{-1}$K${}^{-1}$ to 3.7 Wm${}^{-1}$K${}^{-1}$. In contrast to the size of the flakes, thermal annealing treatment increases $K_{\|}$, with larger changes observed for graphene. This is qualitatively in line with the expectation that annealing improve contacts between adjacent flakes, as also shown qualitatively by the cross-section images (Supplementary Information), by reducing the interfacial scattering and allowing a broader phonon spectrum to be transmitted. This is also reflected in the electrical conductivity, as the sheet resistance of the graphene film decreases after annealing treatment, Figure 3a. Note that values reported in the literature for $K_{\|}$ span from 40 to 140 Wm${}^{-1}$K${}^{-1}$ for very thick laminates made of graphene produced by liquid-phase exfoliation [21,24] and reaches even higher values for graphene produced by electro-chemical or other types of exfoliation methods [43,44]. The highest value reported is 1529 Wm${}^{-1}$K${}^{-1}$ for defect-free graphene, approaching the $K_{\|}$ of graphite of ∼2000 Wm${}^{-1}$K${}^{-1}$ [40]. Finally, in the case of h-BN our value is close to the one reported by Zheng et al. (∼20 Wm${}^{-1}$K${}^{-1}$), for flake size of ∼1 μm [22]. It is important to note that it is challenging to draw conclusions by comparing values in the literature in light of the large role that fabrication methods have on microstructure, defects, interface quality and the resulting transport. However, our results indicate that in the case of inkjet-printed films there is no need to use thick films or large size flakes, as both $K_{\|}$ and $K_{⊥}$ are weakly dependent on those parameters. On the other hand, this also implies that graphene with similar thermal conductivity but very different electrical conductivity can be made very easily by tuning the film thickness or by using post-processing.

The out-of-plane thermal conductivities were found to be remarkably low (0.3–0.5 Wm${}^{-1}$K${}^{-1}$) for all 2D materials investigated, comparable in value to that of glasses [11]. This is striking, considering that conductivities below 1 Wm${}^{-1}$K${}^{-1}$ are typically found in either highly disordered structures such as amorphous Carbon [31] or Selenium [11], or in nanostructures with high atomic mass contrast and interface density [12,14,18,45]. In the present case, along the direction perpendicular to the basal planes, the 2D crystal film structure is not akin to an amorphous structure, nor does it present layers of varying atomic mass contrast, but rather it is more closely related to turbostratic graphite [38]. There are only few reports for graphene films including $K_{⊥}$, with values ranging from 0.25 to 5.5 Wm${}^{-1}$K${}^{-1}$[24,46]. The $K_{⊥}$ for the graphene samples obtained here of ∼0.3 Wm${}^{-1}$K${}^{-1}$ is below 6 Wm${}^{-1}$K${}^{-1}$ for bulk graphite [40] and ∼3 Wm${}^{-1}$K${}^{-1}$ for turbostratic graphite [38]. For comparison, the lowest $K_{⊥}$ for dense layered nanostructures were 0.33 Wm${}^{-1}$K${}^{-1}$ for Au/Si multilayers [18], 0.6 Wm${}^{-1}$K${}^{-1}$ for W/Al${}_{2}$O${}_{3}$ nanolaminates [47] and 0.05 Wm${}^{-1}$K${}^{-1}$ in SnSe${}_{2}$-MoSe${}_{2}$ heterostructures [14]. In the case of dichalcogenide films, turbostratic structures have been shown to yield similar $K_{⊥}$ as the MoS${}_{2}$ sample reported here, with values of 0.3 Wm${}^{-1}$K${}^{-1}$ for sputtered MoS${}_{2}$ [48] and lower values of 0.05 Wm${}^{-1}$K${}^{-1}$ for WSe${}_{2}$ thin films deposited by modulated elemental reactants [12]. The interpretation of the ultralow $K_{⊥}$ in layered materials has typically centered on the dominant role of thermal boundary conductance at phonon-mismatched interfaces. The interpretation of transport in turbostratic dichalcogenide materials has however varied. Muratore et al. interpreted their results on MoS${}_{2}$ by decreasing the bulk conductivity value obtained through the Slack equation through the effect of an additional interface scattering term [48] having scattering length 3–10 nm, as obtained by fitting the experimental data. Erhart et al. interpreted the data of Chiritescu et al. [12] on WSe${}_{2}$ through first principles calculations [17] to conclude that layer stacking disorder and lattice expansion in addition to interface scattering contributed to the low $K_{⊥}$ reported. It is indeed interesting to compare our results with those of Ref. [12], because the films have been grown and the WSe${}_{2}$ nanosheets are expected to have clean interfaces, i.e., no residual solvent or surfactant, although the crystal thickness was limited to <2 nm. In agreement with Ref. [12], the smallest $K_{⊥}$ is not found in the amorphous form, but in a layered structure made of randomly stacked flakes. In comparing our results with Refs. [12,17], and in light of the density functional theory results presented below, we can assert that $K_{⊥}$ in our films are characterized by relatively transmissive interfaces and that the low $K_{⊥}$ is dominated by the small thickness of the flakes.

Our measurements indicate that the thermal conductivity anisotropy $A_{Kth}$ of printed films made of a wide range of 2D materials spans ∼20–40, and this is due to an extremely low $K_{⊥}$. Remarkably, the thermal conductivity of these printed films cannot be tuned by changing the elemental composition of the 2D material and weakly depends on film thickness, size of the flakes and annealing. An overview of the data available in the literature for comparable films is provided in Table 1.

**Table 1:** Summary table of reported thermal conductivity for films made by solution processing. In the table, the following abbreviations are used: rGO for reduced graphene oxide, LPE for liquid-phase exfoliation, ECE for electro-chemical exfoliation, FLG for few-layer graphene, GNP for graphene nano-platelet, IJP for ink-jet printed, NSs for nano-sheets, VF for vacuum filtration.

Materials	Flake Thickness (nm)	Lateral Size (μm)	Thickness (μm)	$K_{\|}$ (Wm${}^{-1}$K${}^{-1}$)	$K_{⊥}$ (Wm${}^{-1}$K${}^{-1}$)	$A_{\mathrm{Kth}}$	Method	Reference
rGO	1.1	—	4.3–12	1100	—	—	2000 ${}^{{}^{\circ}}$C annealed	[49]
rGO	∼1	Avg. area 23 μm^2^	7.5	1390 ± 65	—	—	VF, HI acid reduced	[50]
rGO	∼1	Avg. area 1 μm^2^	—	900 ± 45	—	—	VF, HI acid reduced	[50]
rGO	—	25	—	1434	—	—	Electrospray deposition, 2850 ${}^{{}^{\circ}}$C annealed	[51]
rGO	1–7	108	10	1940 ± 113	—	—	Scraping deposition, compressed and 3000 ${}^{{}^{\circ}}$C annealed	[36]
rGO	<1	>6	0.8	3200	—	—	2850 ${}^{{}^{\circ}}$C annealed, compressed	[52]
rGO	—	—	170	62	0.09	675	1000 ${}^{{}^{\circ}}$C annealed	[23]
rGO + Carbon nanorings	—	—	—	890	5.8	15	VF, in situ growth of CNR (800 ${}^{{}^{\circ}}$C)	[53]
LPE graphene	<10 layers	—	30	110	0.25	440	VF	[24]
LPE graphene	—	0.96–1.24	9–44	40–90	—	—	VF, compressed	[21]
ECE graphene	4	3–4	—	1023	—	—	VF, 2500 ${}^{{}^{\circ}}$C annealed	[43]
ECE graphene	≤8 layers	—	33	674	—	—	VF	[54] ^1^
ECE graphene	∼2.2	>10	5–10	3390	5.5	616	VF	[46]
Fluorinated graphene	0.8–2.3	0.8	10–100	88–242	0.4–22	220–11	Ball milling, VF	[26]
Functionalized FLG	7.35	—	1050	112–123	1.62–1.81	69–68	VF	[27]
GNP	≤10	15	—	178 ± 12	1.28 ± 0.12	139	Microwave exfoliation, VF, 340 ${}^{{}^{\circ}}$C annealed	[28]
GNP	4–5 layers	0.648	30–70	1529	—	—	Ball milling, 2850 ${}^{{}^{\circ}}$C annealed	[44]
IJP graphene	∼2	∼0.2	0.08–0.4	∼12	0.3	∼27–40	LPE, IJP, 150 ${}^{{}^{\circ}}$C annealed	**This work**
hBN laminate	10	1	10–100	20	—	—	LPE, VF	[22]
BN NSs	2.9 ± 0.3	1.8 ± 0.1	10–30	58	3.3	18	Molten alkali-assisted exfoliation, VF, 450 ${}^{{}^{\circ}}$C annealed	[29]
IJP hBN	∼2	∼0.2	0.2–1	∼11	0.5	∼22	LPE, IJP, 150 ${}^{{}^{\circ}}$C annealed	**This work**
MXene (Ti${}_{3}$C${}_{2}$Tx)	—	—	3000	55	—	—	Chemical etching, VF	[55]
IJP MoS${}_{2}$	∼4	∼0.05	0.06–0.25	∼9.5	0.3	∼32	LPE, IJP, 150 ${}^{{}^{\circ}}$C annealed	**This work**

^1^https://doi.org/10.1109/ICEPT.2015.7236587, accessed on 25 October 2022.

## 4. Ab-Initio Modeling

To interpret the present results, we first remark that the phonon mean free paths known for the bulks of the three materials [16,20,56] are much longer that the dimensions of the nanosheets and it is thus possible that the transport within a single flake is approaching the ballistic transport limit. To explore these hypotheses, we use as reference the properties of the three bulk crystalline materials obtained from ab initio calculations based on density functional theory (DFT). Thermal transport conductivities were calculated by using the approach developed by Fugallo [57], by using phonon dispersions and anharmonic three-phonon scattering coefficients computed with density functional theory within the plane-waves and pseudopotential approaches of the Quantum Espresso package [58,59,60]. Computational details are reported in the Supplementary Information. We now examine the problem at various level of complexity.

### 4.1. Ballistic Model and Ideal Interface

To begin, we consider only the transport along the out-of-plane direction. As a first approximation we consider the transport to be entirely ballistic within a single flake and that the thermal resistance is only due to the interfaces among different flakes. Let us consider the system as a stack of planar thin crystal flakes. If the average thickness of one flake is *L* and the conductance associated with the interface is *G* (1/G is the Kapitza resistance [61]), one can easily find that the measured overall film conductivity is K=GL. In this model, the temperature (defined as in the classical textbook examples of electronic ballistic transport [62]) is constant within the thickness of the flakes and the temperature drops only at the interfaces according to J=GΔT, where *J* is the energy flux perpendicular to the interface and ΔT is the temperature drop. Within the Landauer-Buttiker approach [62], the conductance of an ideal interface can be written as a function of the properties of the neighboring bulks:

(1) $$G_{0}=\frac{1}{2}\langle \frac{dn}{dT}\epsilon vs.\rangle .$$

Here, *n* and ϵ are the Bose–Einstein occupation factor and energy of a specific phonon (both characterized by a wavevector k and a branch index ν omitted to simplify the text). *v* is the modulus of the group velocity of that phonon (projected along the direction of transport), and $\langle ...\rangle =1/\left(NV_{c}\right)\sum_{k,\nu }$, where the sum is performed on a grid of *N* wavevectors. $V_{c}$ is the unit-cell volume. Using Equation (1) is equivalent of assuming, as in Ref. [61], that the Kapitza resistance is that of an ideal junction between two phonon reservoirs behaving as black-body emitters [61] or that the interface is totally diffusive [63]. $G_{0}$ from Equation (1) is associated with a transmissivity T=1 for all the carriers and thus we refer to this as the “ideal” interface, keeping in mind that it is associated with a temperature drop and, thus, should not be considered as a perfect grain boundary in which the crystal structure is not disrupted.

Within DFT [58,59], we determined the phonon dispersions of the three bulk crystals and, by means of Equation (1), the ”ideal” conductances $G_{0}$ = 0.247, 0.307, 0.137 Wm${}^{-1}$K${}^{-1}$nm${}^{-1}$ for Graphite, h-BN, and MoS${}_{2}$, respectively. Multiplying these by the measured flake thicknesses we have the purely ballistic conductivity $K_{B}=G_{0}L$ = 0.49, 0.61, 0.51 Wm${}^{-1}$K${}^{-1}$, respectively. Considering the crudeness of the model these numbers are in a remarkable agreement with the measured conductivities (0.30, 0.48, and 0.3 Wm${}^{-1}$K${}^{-1}$, respectively) providing a good hint of the physics at play. To validate this picture we need, first, to quantify at which level the transport can be actually considered ballistic within the flakes. Indeed, while passing through the flakes, phonons undergo other scattering events (this is true even in perfect crystals because of intrinsic anharmonic effects) resulting in a partially diffusive transport regime.

### 4.2. Ballistic vs. Diffusive Transport

The description of an intermediate regime between ballistic and diffusive thermal transport in nanostructured materials is a complex problem not too often discussed (see, e.g., Refs. [63,64,65] and references therein). Here we compare two models, that we will call RS and BT, both based on the ab initio (DFT) phonon properties of the crystals.

Within the “resistors in series” (RS) model, the crystal flake is associated with an intrinsic thermal conductivity $K_{i}$. The resistance of the interfaces and that of the flakes are summed in series. The overall measurable thermal conductivity, expressed as a function of *L*, is then

(2) $$K_{RS}\left(L\right)=\frac{G_{0}LK_{i}}{G_{0}L+K_{i}}.$$

$K_{i}$, which does not depend on *L*, is calculated within the Boltzmann transport Equation (BTE) approach using the single mode relaxation time approximation: $K_{i}=\langle \frac{dn}{dT}\epsilon v^{2}\tau_{i}\rangle$, where $\tau_{i}$ is the intrinsic lifetime of a given phonon calculated by DFT at the lowest anharmonic order (three-phonon scattering) using the approach developed in Ref. [57] (see also Refs. [16,20,56] reporting analogous calculations for the same crystals). Here and in the following, the velocities are always considered as projected along the direction of transport.

The BT model is also based on the BTE, but the phonon lifetime now depends on the flake thickness *L*:

(3) $$K_{BT}\left(L\right)=\langle \frac{dn}{dT}\epsilon v^{2}\tau \left(L\right)\rangle ,\;\;\frac{1}{\tau (L)}=\frac{1}{\tau_{i}}+\frac{2v}{L}F\left(\frac{L}{l}\right),$$

where $l=v\tau_{i}$ is the phonon mean free path and $F\left(x\right)=x\left(1-e^{-x}\right)/\left[2\left(x-1+e^{-x}\right)\right]$, obtained by rewriting the suppression function [64,66] introduced in Equation (2) of Ref. [67]. F(x) is bound between F(0)=1 and F(+∞)=1/2, and the meaning of Equation (3) is straightforward: phonons with mean free path much smaller than *L* (L≫l), behave diffusively and $\tau \left(L\right)∼\tau_{i}$ is purely intrinsic, while those with L≪l behave ballistically and τ(L)∼L/2v does not depend on $\tau_{i}$. Note that Equation (3) is used to describe the conductivity of a system which is not homogeneous in real space. The contribution of a specific phonon (for a given k,ν) is, then, to be interpreted as spatially averaged at the mesoscopic level [64,66,67].

The models RS and BT provide a dependence of the conductivity on *L*, and both have the same limits for the limiting values of *L*: in the diffusive limit the conductivity converges to the bulk intrinsic value ($K_{RS}\left(L\right)≃K_{BT}\left(L\right)≃K_{i}$ for L≫l), while in the ballistic regime (L≪l) the conductivity is that of a series of ideal interfaces ($K_{RS}\left(L\right)≃K_{BT}\left(L\right)≃G_{0}L$ for L≪l). The comparison of the two models, which are based on different principles, can provide an indication of the error that is implicit with these approaches. Most important, the comparison of $K_{RS}\left(L\right)$ and $K_{BT}\left(L\right)$ with the purely ballistic conductivity $K_{B}\left(L\right)=G_{0}L$ at the experimental values of *L* should quantify the importance of the diffusive scattering within a single flake.

Before proceeding, it is interesting to compare τ(L) from Equation (3) with that of the so-called Casimir-Ziman length model (usually written as $\tau^{-1}=\tau_{i}^{-1}+2v/L$), which is commonly used to introduce an extrinsic scattering mechanism in a Boltzmann-type evaluation of lattice thermal conductivity (see, e.g., Ref. [57] and references therein). Although, at first sight, the expression for τ(L) is similar, there are important conceptual differences. The Casimir-Ziman model has been conceived to describe lateral scattering from the lateral borders in, for example, a nanowire: *v* should be the component of the velocity perpendicular to the heat flux and *L* the lateral width of the wire [68]. On the contrary, in the present work *v* is the component of the velocity parallel to the transport direction and *L* is the distance between two barriers at each end of the flake, perpendicular to the transport direction. Moreover, while the Casimir model represents a maximum limit for the scattering reached for rough lateral surfaces (perfectly specular surfaces do not affect the transport) [68], here 2v/L is associated with a barrier having the ideal Landauer-Buttiker conductance $G_{0}$ (perfect transmissivity), which can always be decreased as we will discuss later.

**Figure 4:**
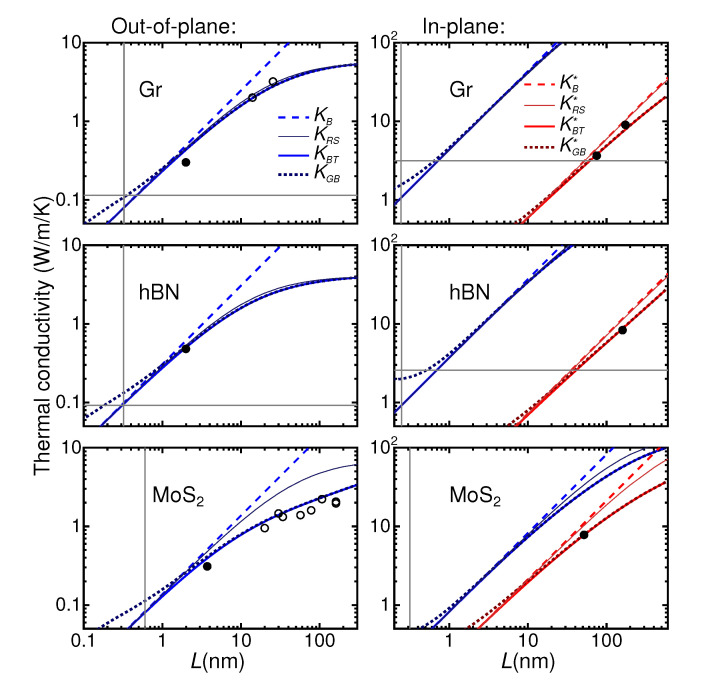
Modelling of the thermal conductivity of assembled flakes with size *L* along two possible transport directions (out-of-plane, $K_{⊥}$, and in-plane, $K_{\|}$) for the three studied materials. Black solid dots are the measurements in this work of non-annealed samples (the abscissa is the average thickness of a single flake within the film), while open dots are from Refs. [20,38,39] (see main text). The lines correspond to various models labeled as B (ballistic), RS (resistance in series), BT (Boltzmann Transport), Generalized Boltzmann (GB), and are obtained assuming an “ideal” (*K*, blue lines) or “dirty” ($K^{*}$, red lines) interface among the flakes. Vertical and horizontal grey lines are defined in the text.

We now compare in Figure 4 the three models: the fully ballistic $K_{B}\left(L\right)=G_{0}L$ model, $K_{RS}\left(L\right)$ and $K_{BT}\left(L\right)$ with the measurements for $K_{⊥}$. For *L* equal to the measured flake thickness (the abscissa of the black dots), $K_{RS}\left(L\right)$ and $K_{BT}\left(L\right)$ do not substantially differ from $K_{B}\left(L\right)$, meaning that the transport is actually predicted to be quasi-ballistic within a single flake. For MoS${}_{2}$, however, the diffusive component of the transport within the flake is not negligible near and above 10 nm of flake thickness. For all the materials, the measured $K_{⊥}$ is not far from the models and we can thus argue that the interface almost behaves as an ideal interface. We cannot claim a quantitative agreement with measurements (in the worst case of graphene, the disagreement is ∼30%), but, given the distribution of flake sizes in the samples and uncertainty in the microstructural flake arrangement, the agreement is overall acceptable.

As a comparison, Figure 4 also reports measurements of $K_{⊥}$ in turbostratic graphite from Refs. [38,39] and MoS${}_{2}$ from Ref. [20] (open symbols) taken in samples having small nanocrystals whose dimensions could be quantified.

### 4.3. Disorder Limit for the BTE

The models discussed so far are meaningful when the conductivity within a flake can be considered as resulting from the sum of single-carriers corresponding to bulk phonons. This assumption is not necessarily acceptable since for an out-of-plane dimension *L* sufficiently small (of the order of the lattice spacing) the system should be considered as disordered. Establishing a minimum value for *L* below which the present treatment becomes meaningless is not a trivial problem and it is remarkable that frameworks for a quantitative answer are possible only thanks to very recent conceptual developments [69,70]. In particular, Ref. [69] provides a more general form for the BTE conductivity that we will call $K_{GB}$ (Equation (12) in Ref. [69]) which is still based on bulk phonon properties but which could be used also to describe disordered systems (the idea that disordered systems can be described starting from bulk phonon properties has been discussed, e.g., in Ref. [71]). In this framework, we can consider the lifetime of a phonon (1/Γ in Equation (12) of Ref. [69]) as an extrinsic parameter which can be tuned to pass from a regime in which the single-phonon BTE picture is acceptable (for large lifetimes, when $K_{BT}$ from standard BTE is not distinguishable from the general form $K_{GB}$ from Ref. [69]) to a regime in which the system should be considered as disordered (for small lifetimes, when $K_{BT}$ and $K_{GB}$ are substantially different). In the present context the phonon lifetimes depend on the thickness of the flake *L*, which can be considered as an external tunable parameter. In Figure 4, we report $K_{GB}\left(L\right)$ obtained by substituting (for every k,ν mode) the lifetime 1/Γ from Equation (12) of Ref. [69] with τ(L) from Equation (3) above. For the three investigated materials, Figure 4, at the measured values of *L*, $K_{BT}\left(L\right)$ and $K_{GB}\left(L\right)$ are not substantially different (above they become indistinguishable) meaning that the single-phonon BTE is still a reasonable approximation. This happens in spite of the fact that, strictly speaking, in none of the three materials studied we can isolate a range for *L* in which $K_{BT}\left(L\right)$ is entirely ballistic (i.e., linear in *L*) and, at the same time, the single phonon BTE can be considered reliable (where $K_{BT}\left(L\right)≃K_{GB}\left(L\right)$), meaning that the transport can never be considered purely ballistic.

### 4.4. Hard Limits of the Phonon Models

As further benchmarks, Figure 4 reports as a vertical line the values of *L* corresponding to the lattice spacing along the transport direction. Figure 4 also reports as horizontal lines the conductivities obtained by substituting the phonon lifetime τ in Equation (Figure 3) with half of the phonon period (τ=πℏ/ϵ for every k,ν mode). The values obtained for MoS${}_{2}$ (0.03 and 0.4 Wm${}^{-1}$K${}^{-1}$ for out-of- and in-plane) are out of scale. The idea, which has been already employed to discuss related problems [12,72], is that of the minimum conductivity model [73], which provides a lower limit to the lattice thermal conductivity of a material. On the left side of the vertical lines and below the horizontal ones, the present models are meaningless.

### 4.5. Ideal vs. Dirty Interfaces

We now discuss the in-plane transport. The models discussed so far are based on the concept of “ideal” interfaces, meaning, in the language of the Landauer-Buttiker approach, that the interface transmissivity T=1 for every phonon. If we apply the same models to the in-plane transport $K_{\|}$ (right panels of Figure 4) the agreement is very poor, providing a conductivity much larger than the measured one (up to almost two orders of magnitude for graphene). The explanation of this in-plane out-of-plane asymmetric behavior is to be found in the geometry of the system. Indeed, we are studying very thin and relatively wide flakes obtained from lamellar materials. From scanning electron microscopy, the flakes appear to stick one on the top of the other with a relatively flat surface. On the contrary, the flakes’ lateral geometry is not well defined and (unless we conceive the in-plane arrangements of the flakes as a tilework) the contact between two adjacent borders is more disturbed, possibly presenting small void regions.

A more suitable form for the interface conductance is then

(4) $$G^{*}=\frac{1}{2}\langle \frac{dn}{dT}\epsilon vs.T\rangle ,$$

where T≤1 is the transmission associated with a given phonon k,ν. We consider $T=e^{-x^{2}}$, where $x=\epsilon /E_{c}$, $E_{c}$ a cut-off energy characterizing the interface. A simple extension of the RS and BT models is obtained by substituting $G_{0}$ with $G^{*}$ in Equation (2) and *L* with TL in Equation (3). The corresponding $K_{RS}^{*}\left(L\right)$ and $K_{BT}^{*}\left(L\right)$ have the limits $K_{RS}^{*}\left(L\right)≃K_{BT}^{*}\left(L\right)≃K_{i}$ for L≫l and $K_{RS}^{*}\left(L\right)≃K_{BT}^{*}\left(L\right)≃K_{B}^{*}\left(L\right)=G^{*}L$ for L≪l. Considering $E_{c}$ as a fitting parameter, the measured values for $K_{\|}$ are reproduced with $E_{c}$ = 11, 11, 17 meV (for graphene, h-BN and MoS${}_{2}$, respectively), which cut off an important part of the phonon spectrum (see Supplementary Information). The analogous fit for the measured $K_{⊥}$, gives higher $E_{c}$∼44, 350, 55 meV, respectively, mildly affecting the conductance. The calculated $K_{\|}$ curves are reported as red lines in the right panels of Figure 4, while the analogous $K_{⊥}$ are not shown since they almost superimpose with the “ideal” lines already present.

Note that within this picture the measured $K_{⊥}$ of hBN seems to be better than that of graphene and MoS${}_{2}$ (this corresponds to the fact that in Figure 4), left-panels, the experimental data for hBN are much more similar to the corresponding ideal-interface calculations). We can speculate that, because of the presence of a stronger ionic character of the inter-plane bonding, the hBN flakes are more tightly bound. The difference can also be attributed to the amount of residual stabilizer affecting the interface transport, as from our XPS data reported in the Supplementary Information it is <10 % for graphene and MoS${}_{2}$, and <3 % for hBN.

## 5. Conclusions

Concluding, the measured conductivities are compatible with the presence of relatively clean flakes: the transport is quasi-ballistic within a single flake and the thermal resistance is essentially due to the interfaces among different flakes. In spite of $K_{⊥}$ being ultra-low, $K_{⊥}$ is explained by relatively clean, high-transmissivity interfaces, and a model based on the ”ideal” Landauer-Buttiker interface conductance gives a qualitatively good result for graphene, h-BN and MoS${}_{2}$. On the contrary, $K_{\|}$ is much smaller than predicted by such an “ideal” model, and measurements can be explained only by invoking an interface transmissivity cutting off an important part of the phonon carriers. This anisotropic behavior (good out-of-plane transmissivity vs. bad in-plane transmissivity) is compatible with the intrinsic geometry of the system consisting on relatively thin and flat flakes sticking on one another. Within this picture, the effect of the annealing on the samples is that removing interstitial water molecules or altering the bonding between flakes improves the interface conductance. Thus, we argue that the use of different chemistry leading to a different kind of inter-flake bonding could be exploited as a means to increase the lateral thermal contact conductance among flakes and/or diminish the one along the out-of-plane direction (which in this work is almost ideal). Phonon modal mismatch across flakes of different materials (as in heterogeneously layered 2D crystals) can also lower the transmissivity and further reduce $K_{⊥}$. All these effects would lead to a further increase of the thermal conduction anisotropy.

## Data Availability

The data presented in this study are available on reasonable request from the corresponding author.

## Supplementary Materials

Supplementary information can be downloaded at: https://www.mdpi.com/article/10.3390/nano12213861/s1. The supplement contains information on: ink preparation and printing, 2D crystal film characterization (transmission electron microscopy, atomic force microscopy, Raman microscopy, profilometry, scanning electron microscopy, electrical characterization, residual PS1), additional details and sources of error for FDTR measurements of thermal conductivity, and additional details on density functional theory calculations. References [74,75,76,77,78,79,80,81,82,83,84,85,86,87] are cited in the supplementary materials.
