# Supplementary material for: Anisotropic Thermal Conductivity of Inkjet-Printed 2D Crystal Films: Role of the Microstructure and Interfaces
Source: Nanomaterials (Basel). 2022 Nov 1;12(21):3861. doi: 10.3390/nano12213861 (PMC9654414; doi:10.3390/nano12213861)
Supplement: Supplementary file 1 [file nanomaterials-12-03861-s001.zip › nanomaterials-1979167-supplementary.pdf]

Anisotropic thermal conductivity of inkjet-printed 2D crystal films: role of the microstructure and interfaces

Mizanur Rahman[†], Khaled Parvez[2], Giorgia Fugallo[3], Chaochao Dun[4], Oliver Read[2], Adriana Alieva[2], Jeffrey J. Urban[4], Michele Lazzeri[5], Cinzia Casiraghi[2], Simone Pisana[1,6,]*

[1] Department of Physics and Astronomy, York University, Toronto, Canada

[2] School of Chemistry, Manchester University, Manchester, UK

[3] Université de Nantes, CNRS UMR 6607, Nantes, France

[4] Lawrence Berkeley National Laboratory, Berkeley, California, USA

[5] Sorbonne Université, CNRS UMR 7590, Paris, France

[6] Department of Electrical Engineering and Computer Science, York University, Toronto, Canada

[*] pisana@yorku.ca

Supplementary information

1. Ink Preparation and Printing

2. 2D Crystal Film Characterization

- **2.1 Transmission Electron Microscopy**
- **2.2 Atomic Force Microscopy**
- **2.3 Raman spectroscopy**
- **2.4 Profilometry**
- **2.5 Scanning Electron Microscopy**
- **2.6 Electrical Characterization**
- **2.7 Residual PS1**

3. Additional details and sources of error for FDTR measurements of thermal conductivity

4. Additional details on density functional theory calculations

1. Ink Preparation and Printing

Bulk graphite (Sigma Aldrich, 100+ mesh), bulk boron nitride (Sigma Aldrich, >1μm) and bulk MoS2 (Sigma Aldrich) powders were used to prepare the inks. The bulk powders were dispersed in DI water at a concentration of 3 mg mL^{-1} and 1-pyrenesulfonic acid sodium salt (PS1), purchased from Sigma Aldrich (purity ≥97%), was added at a concentration of 1 mg mL^{-1}. The graphite, boron nitride and MoS_2 dispersions were sonicated for 120 h using a 300 W Hilsonic HS 1900/Hilsonic FMG 600 bath sonicator at 15 °C. The resultant dispersions were centrifuged at 3500 rpm (g factor = 903) for 20 min using a Sigma 1-14K refrigerated centrifuge in order to separate out and discard the residual bulk and non-exfoliated flakes. The remaining supernatant was centrifuge twice at 16,000 rpm for 60 min to remove excess PS1 from the dispersion. After washing, the precipitate was re-dispersed in the printing solvent, made as described in Ref. [S1]. Another graphene ink with smaller flake size was prepared using a bath sonicator for 120 h followed by a probe-sonicator (Fisher Scientific, FB50) for 3.5 h. Diameter of the probe was 1/8 inch and the ultrasonication frequency was 20 kHz. In order to keep a constant temperature, the vessel was placed in an ice bath during sonication. After sonication, the graphene dispersion was centrifuged and re-dispersed in the printing solvent following the procedure described above.

The concentrations of the resultant inks were assessed using a Varian Cary 5000 UV-Vis spectrometer and the Beer-Lambert law, with extinction coefficients of 2460 L gm^{-1} (at 660 nm) [S2] 1000 L gm^{-1} (at 550 nm) and 3400 L gm^{-1} (at 672 nm) [S3], respectively. The inks used for printing were diluted to a concentration of ~2 mg mL^{-1}.

A Dimatix DMP-2800 inkjet printer (purchased from Fujifilm Dimatix) was used to print the 2D crystal inks (i.e. graphene, h-BN and MoS_2) onto SiO_2/Si substrates. Prior to inkjet printing, the SiO_2/Si substrates were cleaned with both acetone and 2-propanol for 15 min each in an ultrasonic bath, followed by drying with N_2. The nozzle plate of the printer cartridge consists of a single row of 16 nozzles of 21 μm diameter spaced between 254 μm apart with typical drop volume of 10 pL. The 2D crystal inks were printed at a voltage of ~25 V using a jetting frequency of 5 kHz and with a drop spacing of 35 μm, while maintaining the substrate platen temperature at 40 C, to facilitate the rapid drying of the ink.

2. 2D Crystal Film Characterization

2.1 Transmission Electron Microscopy

Representative transmission electron microscopy (TEM) pictures of the 2D crystal inks used in this work are shown in Figure S1. Inks were dispersed on an ultrathin lacey carbon support film (copper) and imaged in a Joel 2100-F. The microscope is aligned for beam energy at 200 keV. HRTEM studies revealed the excellent quality of the exfoliated 2D graphene (top), hBN (middle) and MoS2 (bottom) flakes.

The figure shows flakes having lateral sizes compatible with the analysis of AFM data below. The flakes are highly crystalline as shown in the high-resolution images. The selected area diffraction obtained from the high-resolution images show the characteristic six-fold symmetry expected for these materials. By counting the number of layers visible in flakes imaged in cross-section, it was determined that the great majority of the flakes consisted of 4-7 layers [S4].

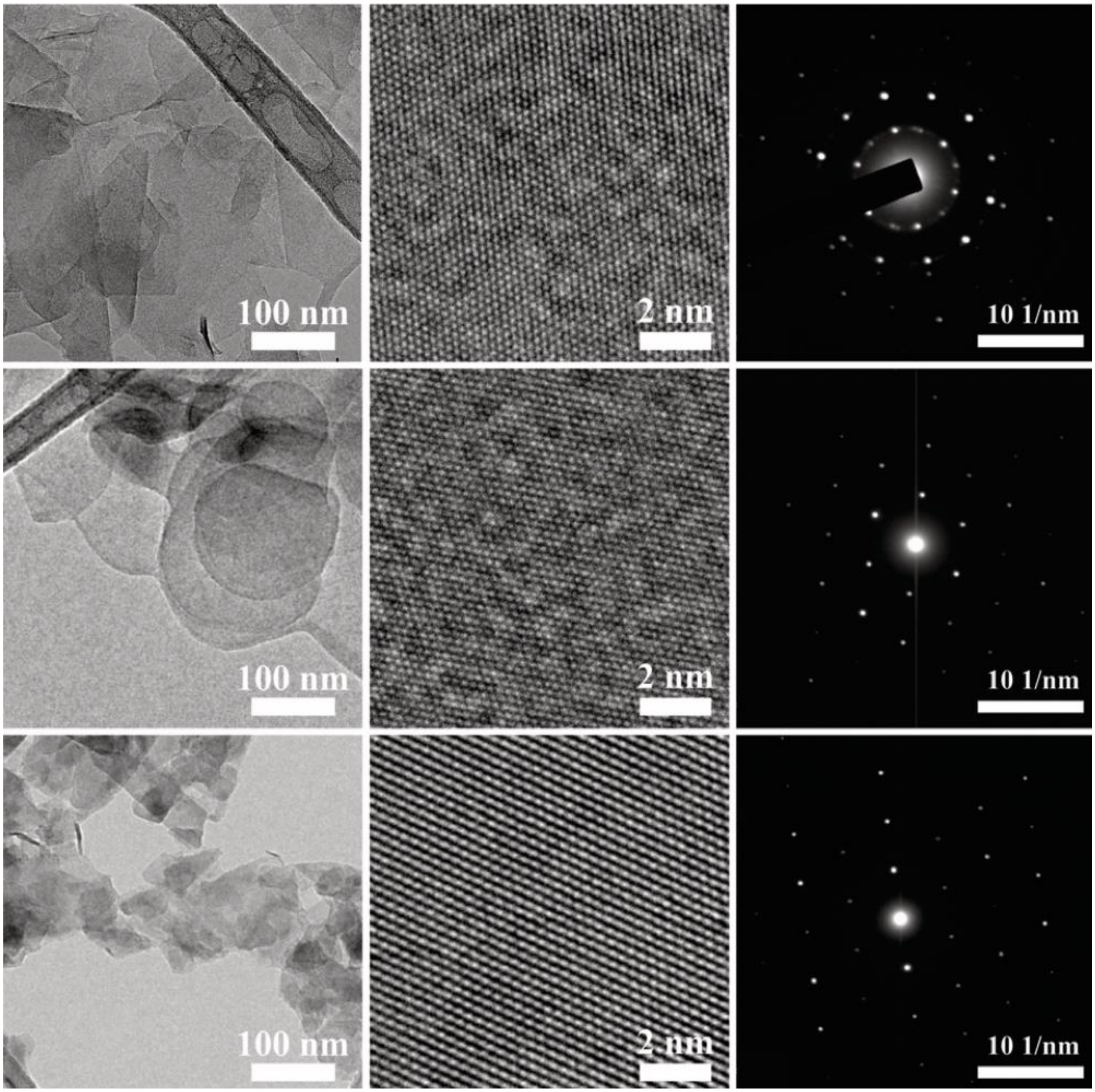

Figure S1. TEM micrographs of graphene (top), hBN (middle) and MoS_2 (bottom) inks. The left panel are lower-magnification images, the right panels are selective-area diffraction images obtained in the areas imaged in the middle panels.

2.2 Atomic Force Microscopy

Atomic force microscopy (AFM) was carried out on dilute printed films on Si/SiO_2 substrates in order to obtain statistical lateral size and thickness distribution of the flakes. As mentioned in the main text, the flake thickness obtained in this way includes the presence of residual stabilizer on its surface. Precise estimation of the number of layers can be obtained only with transmission electron microscopy. The AFM characterization was carried out for both typical samples created by 120h bath sonication (Figure S2), as well as smaller graphene flakes created by an additional 3.5h tip sonication (Figure S3).

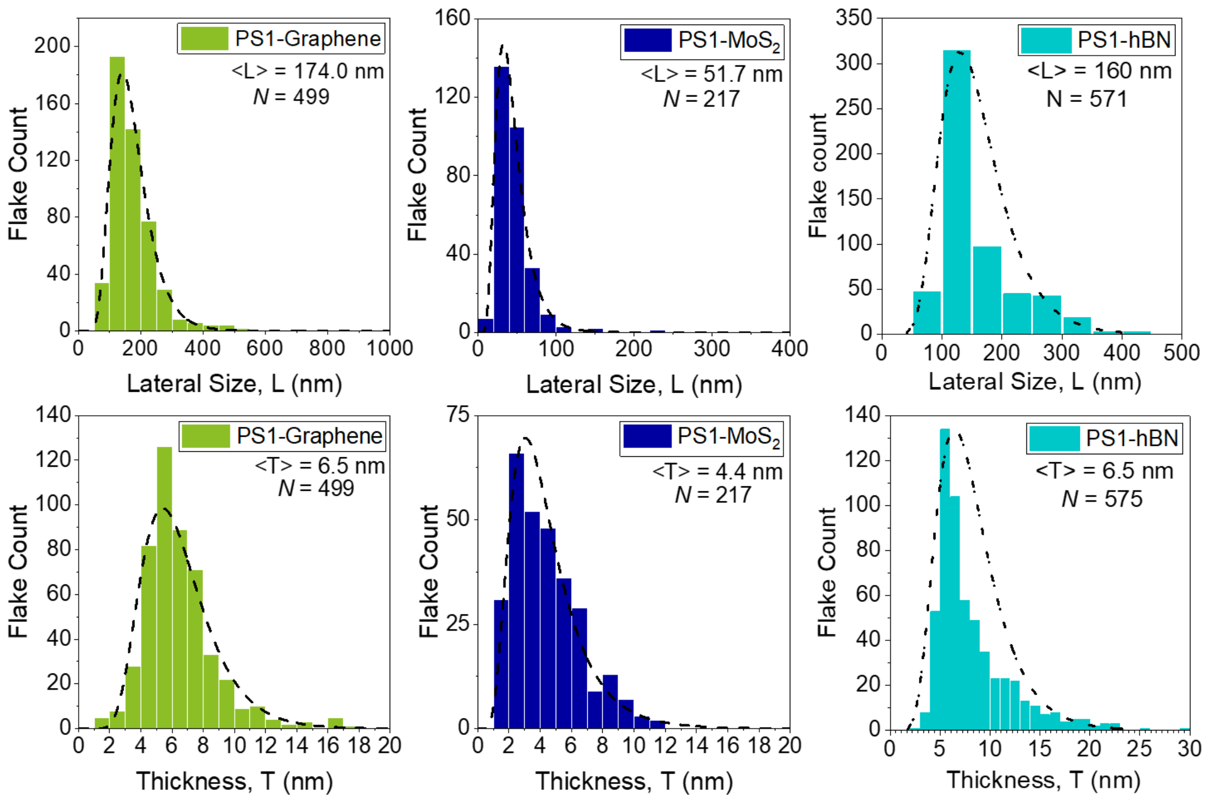

Figure S2. Histogram of lateral size (L) and thickness (T) distributions of graphene (green), MoS_2 (blue), and hBN (teal) flakes derived by AFM. N is the number of flakes measured. The statistical averages of size and thickness are indicated in each panel.

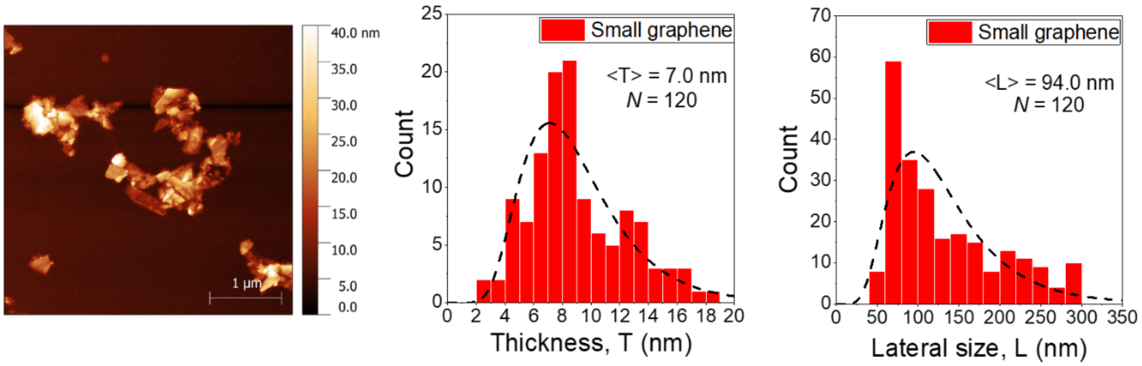

Figure S3. (left panel) Typical AFM topography of smaller graphene flakes obtained by additional 3.5h tip-sonication (see Methods in the main text). (center and right panels) Histograms of lateral size (L) and thickness (T) distributions of the resulting smaller graphene flakes. The statistical averages and sample size are indicated in each panel.

2.3 Raman spectroscopy

Figure S4 shows representative Raman spectra measured on individual flakes drop-cast on silicon substrate, confirming the typical features of these 2D materials. The Raman spectra show the typical features of graphene, MoS_2 and hBN. In the case of graphene, the D peak is typical of nanosheets produced by liquid-phase exfoliation, as their size is smaller or comparable to that of the laser spot size (~500nm).

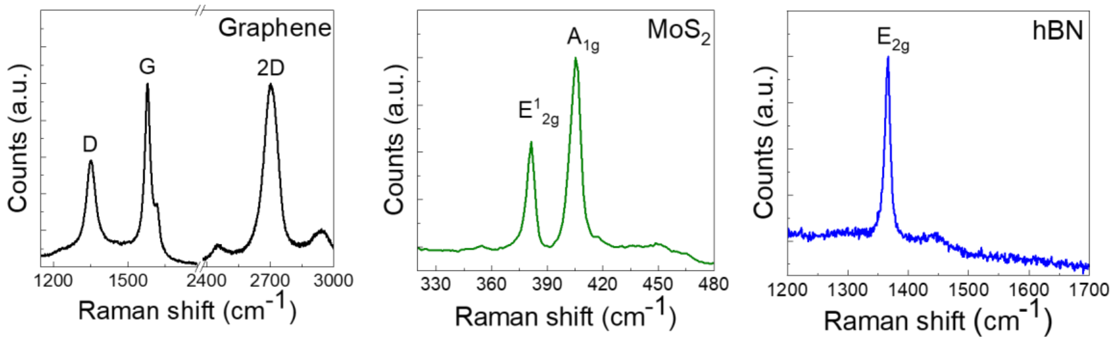

Figure S4. Raman spectra of graphene (left), MoS_2 (center) and hBN flakes (right).

2.4 Profilometry

Profilometry was performed on the printed film to determine thickness and roughness. Figure S5 shows an optical picture taken with the microscope of the printed lines made with different 2D material inks, and the cross-sectional profilometry of the lines for graphene, MoS_2 and hBN printed films. Figure S6 summarizes the data showing the thickness vs number of printed passes. A small decrease in the thickness is observed as a result of desorption of some of the residual stabilizer or additive used for printing.

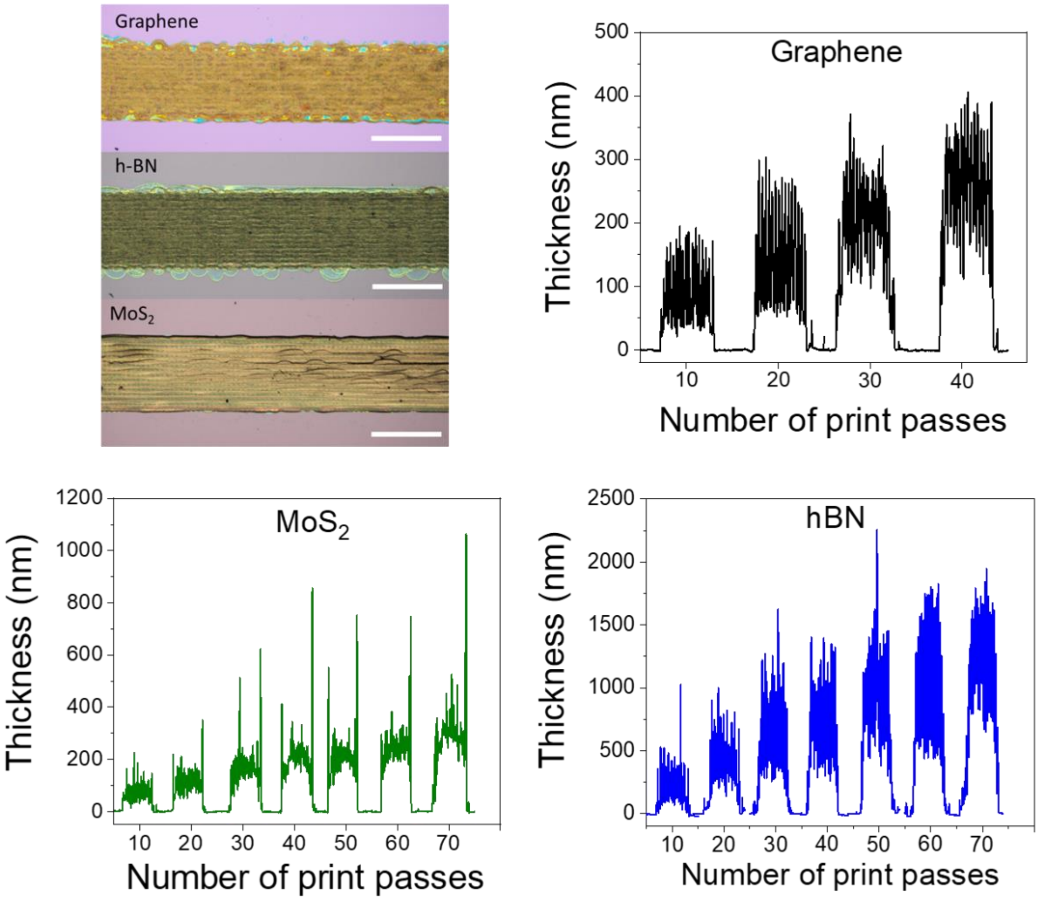

Figure S5. (top-left) Microscope images of inkjet printed 2D crystal films of graphene, hBN and MoS_2. The scale bar is 500 µm. The other panels show profilometry data of the films, indicating thickness as function of print passes.

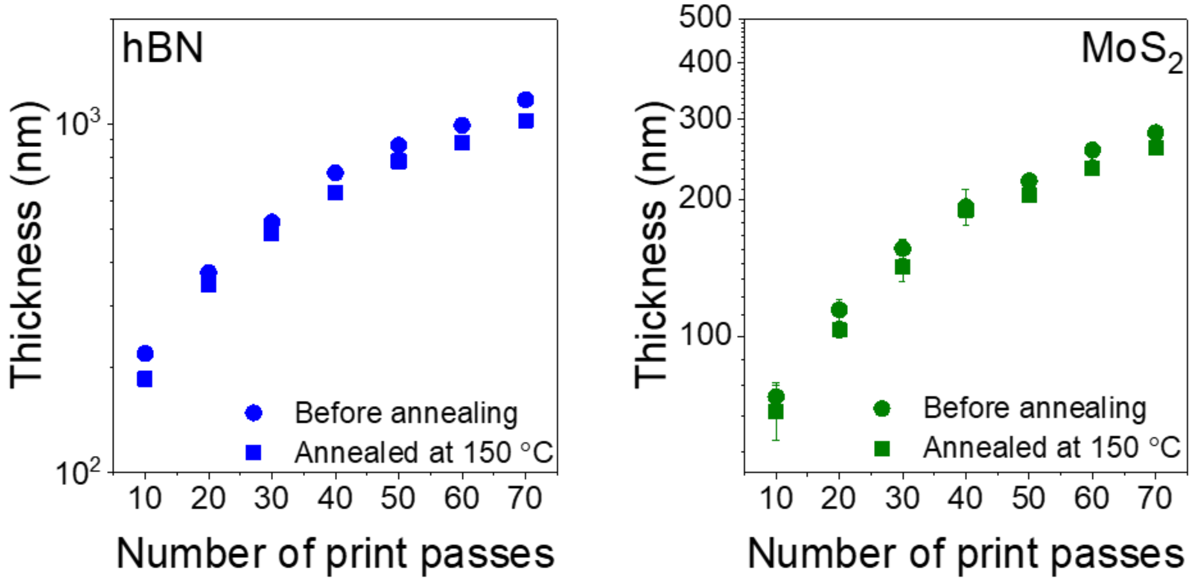

Figure S6. Profilometry data showing the film thickness as function of printing passes before and after annealing for hBN (left) and MoS_2 (right).

2.5 Scanning electron microscopy

Scanning electron microscopy (SEM) was carried out to investigate the microstructure of the inkjet-printed films. Prior to SEM imaging, the samples were briefly dipped in liquid nitrogen and then fractured to expose the film cross-section. While this procedure was adopted to minimize disruption of the film morphology prior to imaging, we cannot guarantee that the fracturing itself has not disturbed the film structure, and therefore quantitative assessment is difficult. Note that the images below were taken for films substantially thicker than the ones in this study, and therefore provide an upper bound to the structural disorder, as thinner films more easily conform to the flat substrate. Figure S7a shows sample cross-sectional images from h-BN films before and after annealing treatment. By comparing the pristine and annealed film, one can see that annealing produces a somewhat improved alignment of the flakes. This gives rise a slight improvement in the in plane thermal conductivity (from 8 to 10 W/(m·K), showing that these morphological parameters have very small effect of the in-plane conductivity. We remark that the film morphology obtained by inkjet printing is distinct from that produced by vacuum filtration (Figure S7b), which is mostly used in the works in the literature reporting anisotropy ratio. Vacuum filtration gives rise to very thick films whose morphology is changing over the cross section of the film.

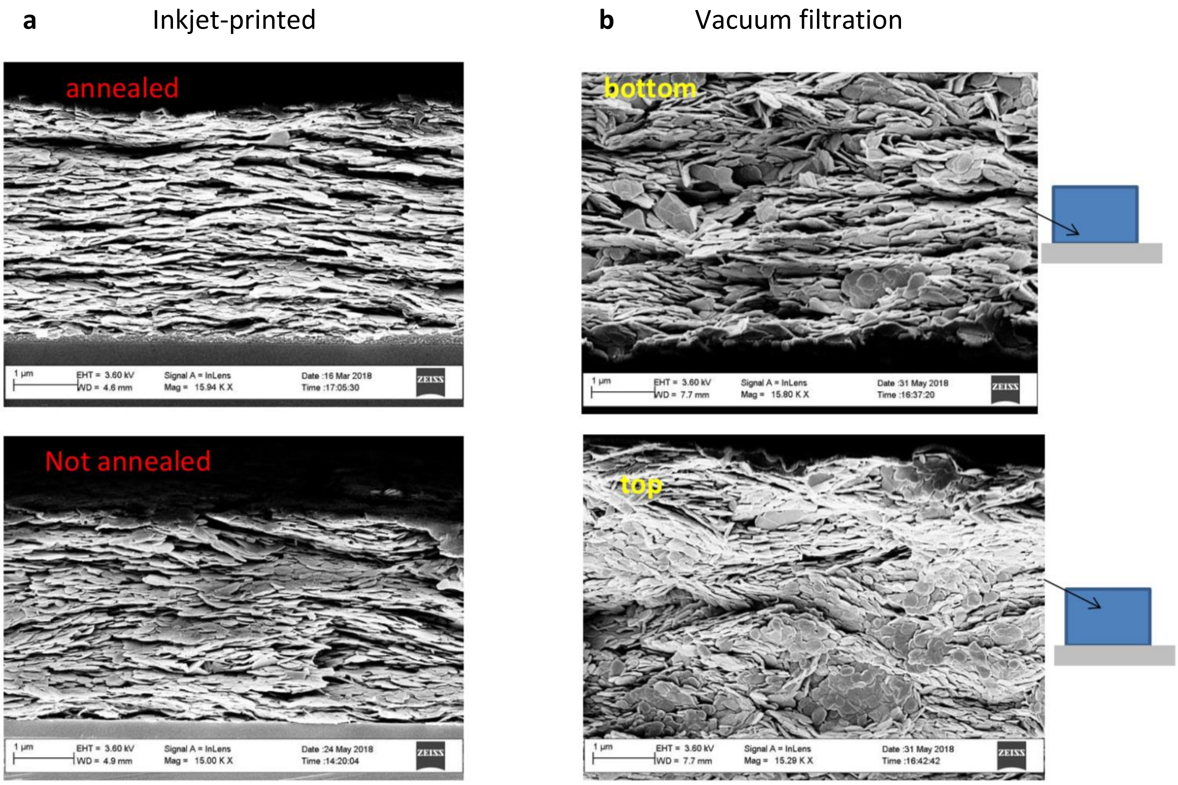

Figure S7. Cross-sectional SEM imaging after cryo-fracturing (**a**) inkjet-printed h-BN films before and after annealing, and (**b**) vacuum-filtered h-BN films at different positions of the thick film structure.

2.6 Electrical characterization

The electrical properties of the printed graphene were measured with two contact probes using an Agilent B1500 probe station. The sheet resistance (R_s) values were calculated from the current-voltage profiles using the equation: $R_s = R \times W/L$, where R, W and L are the resistance, width and the length of the printed patterns, respectively. The thickness of the of the printed patterns were measured by using a Bruker Dektak XT surface profiler. The results of the electrical characterization are presented in the main text.

2.7 Residual PS1

The residual PS1 in the 2D crystal films has been determined by XPS analysis as reported separately in Ref S4, and summarized in Table S1 below.

Table S1. Upper-bound residual PS1 concentrations for different 2D crystal films as determined by XPS analysis.

	**Graphene**	**h-BN**	**MoS_2**
Residual PS1 (wt. %)	< 10	< 3	< 10

3. Additional details for FDTR measurements of thermal conductivity and sources of error

A detailed description of our FDTR system and fitting procedure to the thermal model has been published elsewhere [S5, S6]. The samples, covered by the 50 nm thick Al transducer layer, are modeled as a multilayer of finite-thickness materials and the interfaces between them, as follows: Al/$G_{Al\text{-}Film}$/Film/$G_{Film\text{-}SiO2}$/SiO_2/$G_{SiO2\text{-}Si}$/Si. The first layer is the Al transducer, Film is the 2D crystal film studied here, SiO_2 is the thermal oxide and Si is the substrate. The interface between each layer is characterized by the thermal boundary conductance across the respective layers, i.e. $G_{Al\text{-}Film}$ is the thermal boundary conductance at the Al-film interface, $G_{Film\text{-}SiO2}$ is the thermal boundary conductance of the film-SiO_2 interface, and $G_{SiO2\text{-}Si}$ is the thermal boundary conductance for the SiO_2-Si interface.

Each finite layer in the model is characterized by its thickness, (predetermined by AFM or optical profilometry), anisotropic thermal conductivity values $K_{\perp}$ and $K_{\parallel}$, and volumetric heat capacity C. $K_{\perp}$ and $K_{\parallel}$ of the 2D crystal films were fitting variables, and all the other parameters were taken as known constants. Interfaces were modeled by their respective values of thermal boundary conductance. The value for $G_{Al\text{-}Film}$ was measured for several representative films and then kept constant in order to reduce error propagation, since the value of $G_{Al\text{-}Film}$ was not found to vary appreciably among different samples of the same film material. The Al, SiO_2 and Si layers were assumed to be isotropic ($K_{\perp} = K_{\parallel}$). Their thermal conductivities were predetermined via separate measurements: for the Al layer, the conductivity was determined by 4-point probe electrical conductivity measurement and applying the Wiedemann-Franz law; for the SiO_2 and Si layers, separate FDTR measurements were performed with a top Al layer. The volumetric heat capacities of Al, SiO_2 and Si are well known and were taken from the literature. Typical parameters used in the thermal model are summarized in Table S2 below.

The volumetric heat capacity of the 2D crystal film could be a source of systematic error. In our model, C of films is considered to be similar to the bulk value for that material. This in reality could be smaller if the film is porous. The error introduced in the reported values of thermal conductivity due to the assumed value of C is comparatively small. For example, for the Graphene film samples we take C = 1.59 x 10^6 J/m^{3}K as in graphite. For a representative measurement yielding $K_{\parallel}$ = 9.1 W/mK and $K_{\perp}$ = 0.33 W/mK, lowering the assumed value of C to 1.3 x 10^6 J/m^{3}K changes the fitted values to 8 and 0.38 W/mK, respectively. The influence of C on the derived thermal conductivities are similar in other materials. We note that a film having C reduced to the value of 1.3 x 10^6 J/m^{3}K used in this example would be associated with a graphite film having a density 86.7% of the bulk value (13.3 % of the volume being voids).

Representative results showing FDTR data and fits to the thermal model are shown in Figures S8-S14. Figure S15 summarized the FDTR data for all samples measured as function of film thickness and annealing condition.

Table S2. Typical thermal model parameters used to analyze FDTR data. Most parameters were assumed constant, whose value was determined independently as indicated or taken from the literature. In most fits, only the $K_\perp$ and $K_\parallel$ values for the 2D crystal films were adjustable.

Layer	Value	Source
Al	$K_\perp$ = $K_\parallel$ = 50 W/mK	4-point probe
	C = 2.42 x 10^6 J/m^3K	[S7]
	t = 40 – 60 nm	Optical profilometry and picosecond acoustics
Al-Film Interface	G = 40 x 10^6 W/m^2K	FDTR fits
Film	$K_\perp$ = 0.3 W/mK<br>$K_\parallel$ = 10 W/mK	FDTR fits
	C = 1.59 x 10^6 J/m^3K [graphene]	[S8]
	C = 1.84 x 10^6 J/m^3K [h-BN]	[S9]
	C = 1.89 x 10^6 J/m^3K [MoS_2]	[S10]
	t = 60 – 1,200 nm	Profilometry
Film-SiO_2 Interface	G = 20 – 40 x 10^6 W/m^2K	[S11-S12]
SiO_2	$K_\perp$ = $K_\parallel$ = 1.32 W/mK	[S13]
	C = 1.59 x 10^6 J/m^3K	[S13]
	t = 250 – 300 nm	FDTR fits on areas without film
Si	$K_\perp$ = $K_\parallel$ = 145 W/mK	[S13]
	C = 1.64 x 10^6 J/m^3K	[S13]
	t = infinite	Boundary condition

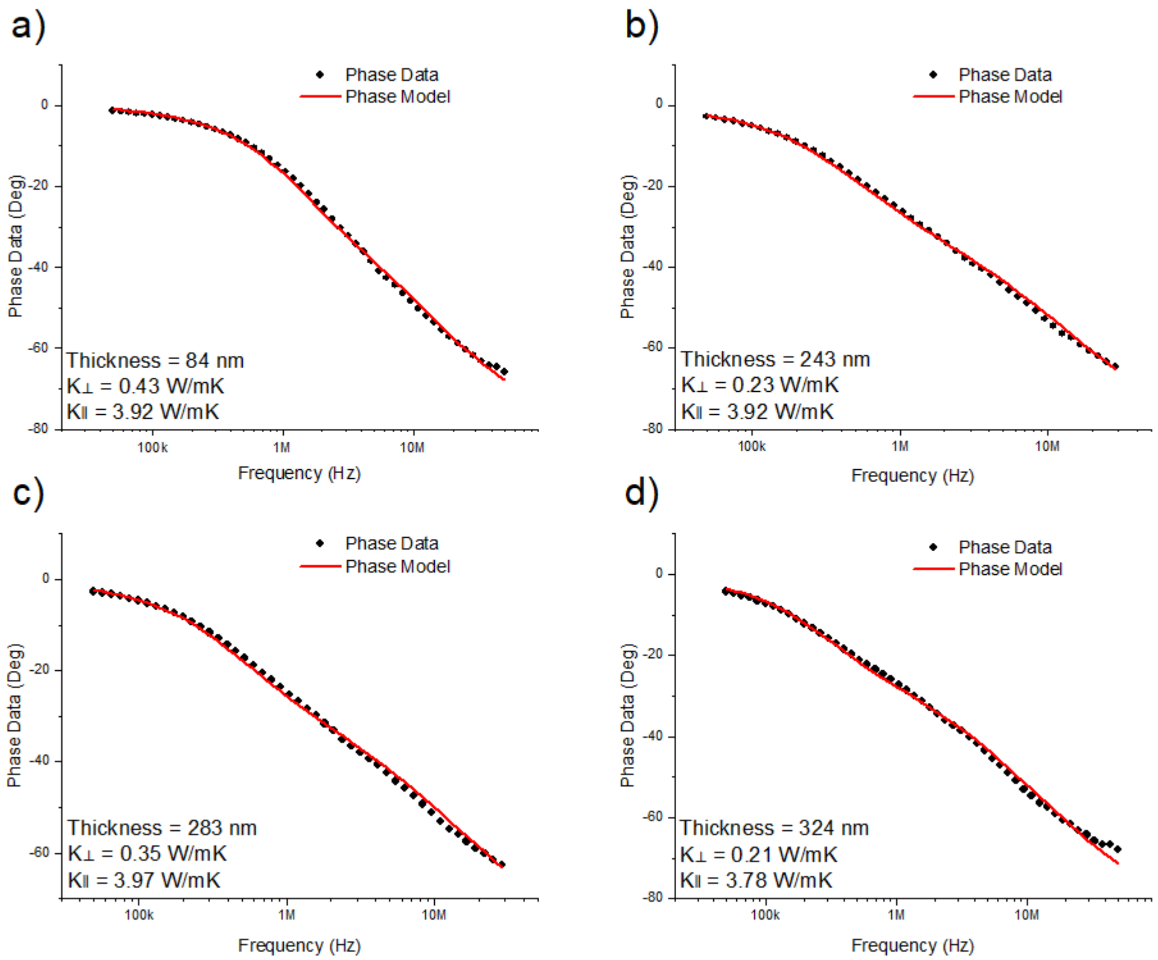

Figure S8. Representative FDTR measurement and fit to the thermal model for the small-flake (longer sonication) pristine graphene film. The film thickness and the extracted thermal parameters are indicated in each panel.

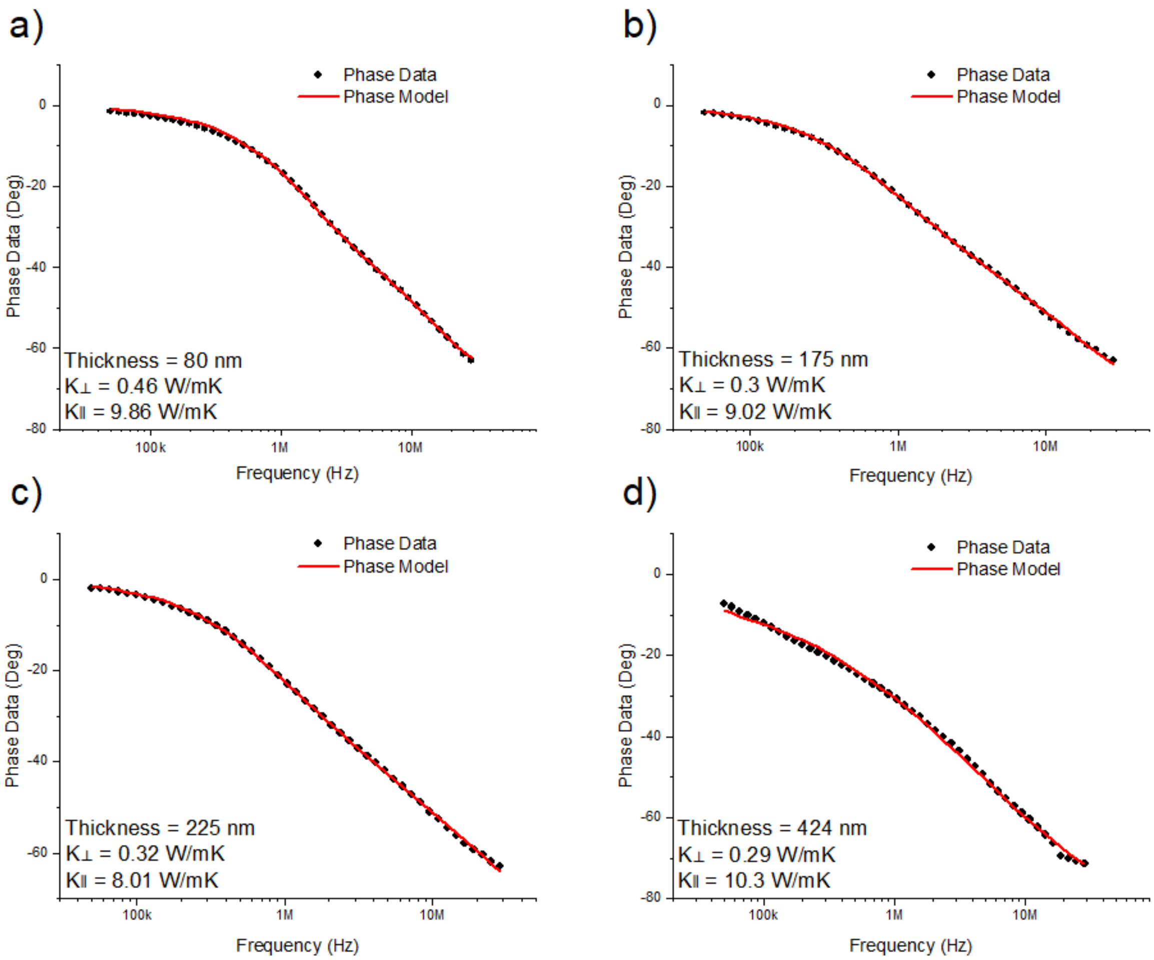

Figure S9 Representative FDTR measurement and fit to the thermal model for the pristine graphene film. The film thickness and the extracted thermal parameters are indicated in each panel.

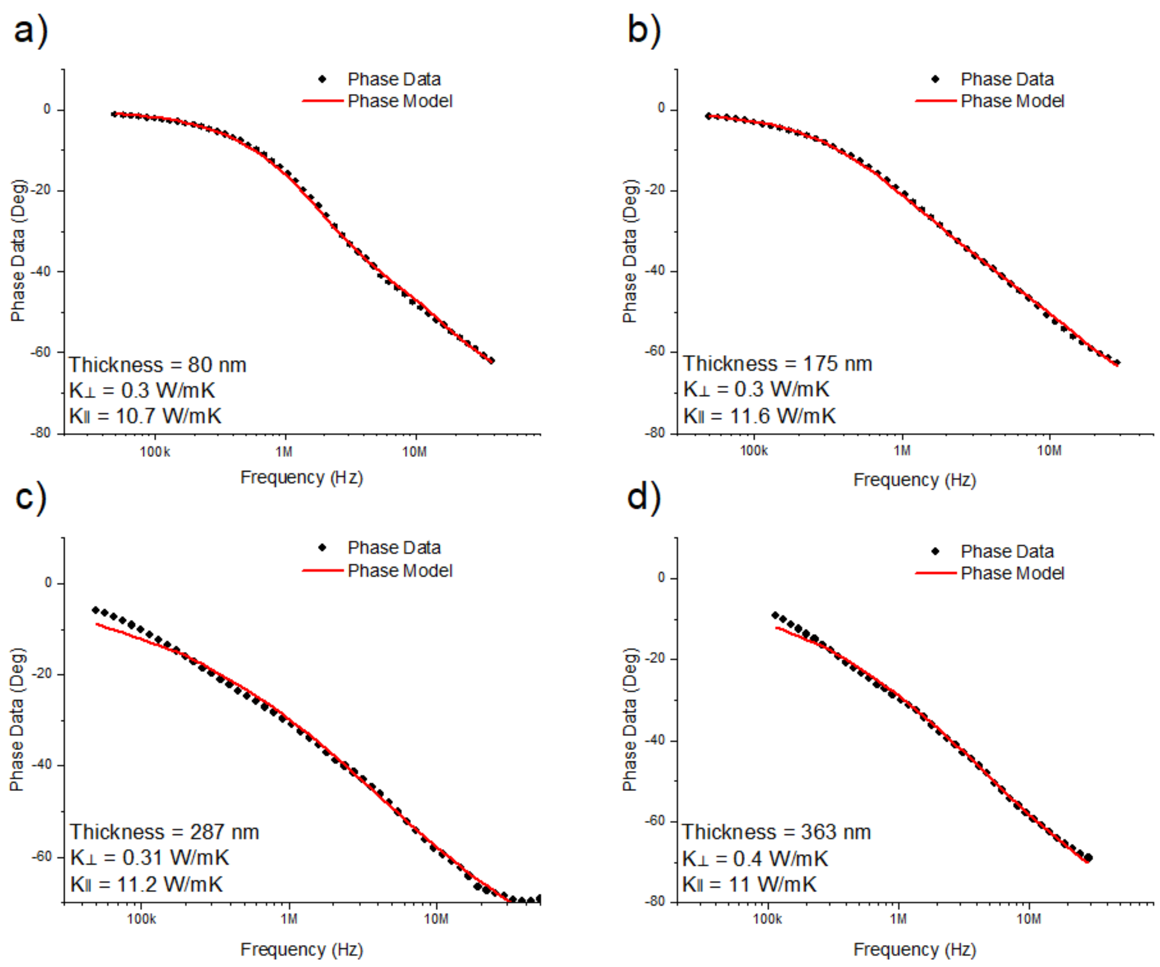

Figure S10. Representative FDTR measurement and fit to the thermal model for the annealed graphene film. The film thickness and the extracted thermal parameters are indicated in each panel.

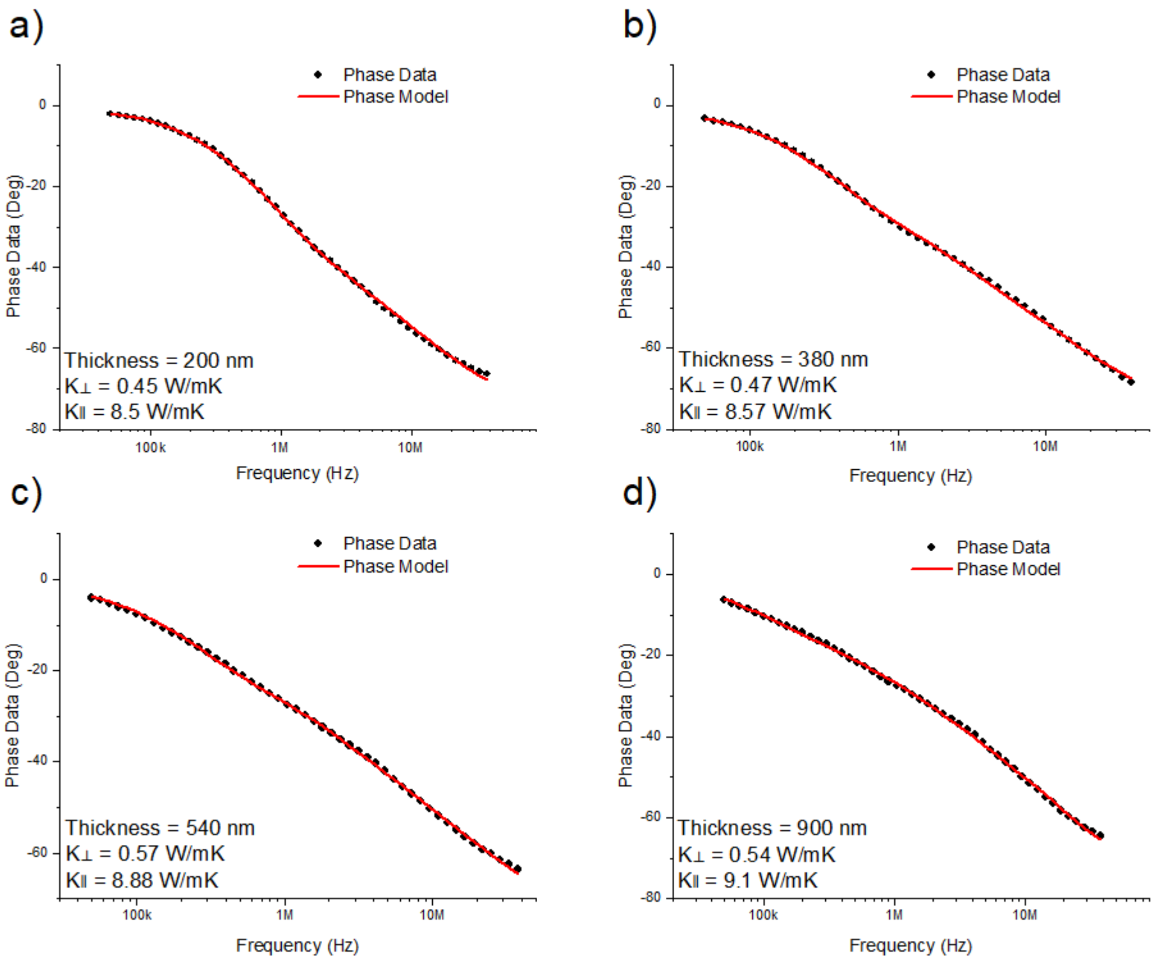

Figure S11. Representative FDTR measurement and fit to the thermal model for the pristine h-BN film. The film thickness and the extracted thermal parameters are indicated in each panel.

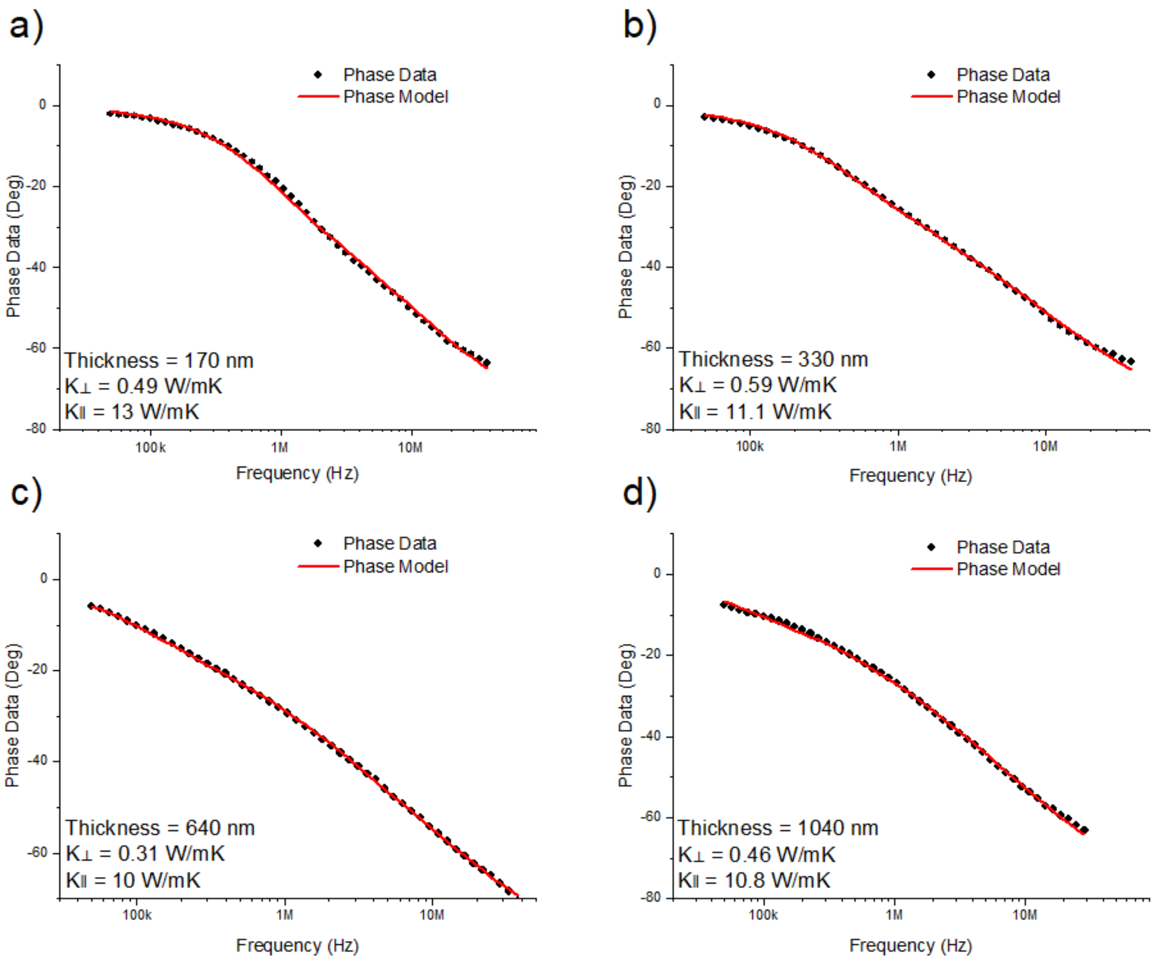

Figure S12. Representative FDTR measurement and fit to the thermal model for the annealed h-BN film. The film thickness and the extracted thermal parameters are indicated in each panel.

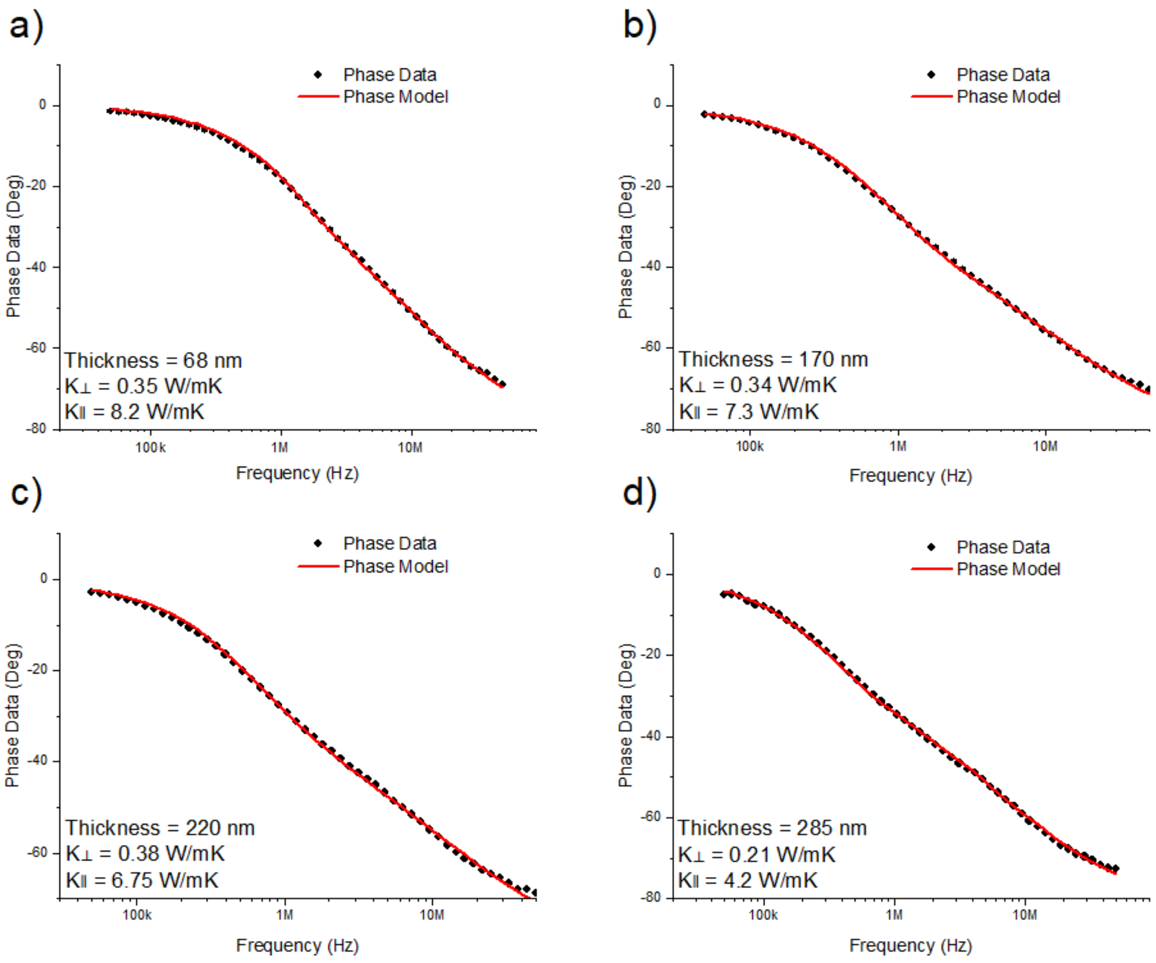

Figure S13. Representative FDTR measurement and fit to the thermal model for the pristine MoS_2 film. The film thickness and the extracted thermal parameters are indicated in each panel.

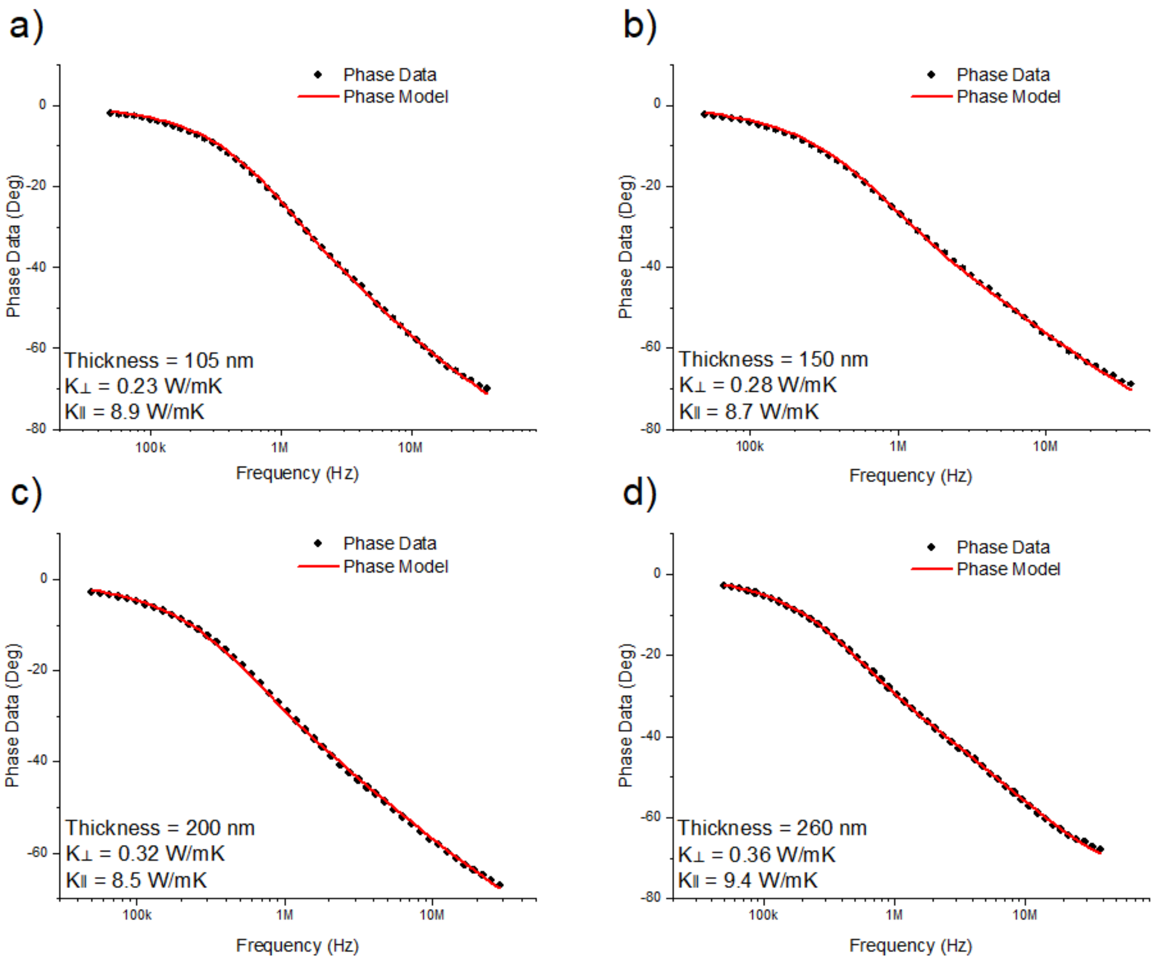

Figure S14. Representative FDTR measurement and fit to the thermal model for the annealed MoS_2 film. The film thickness and the extracted thermal parameters are indicated in each panel.

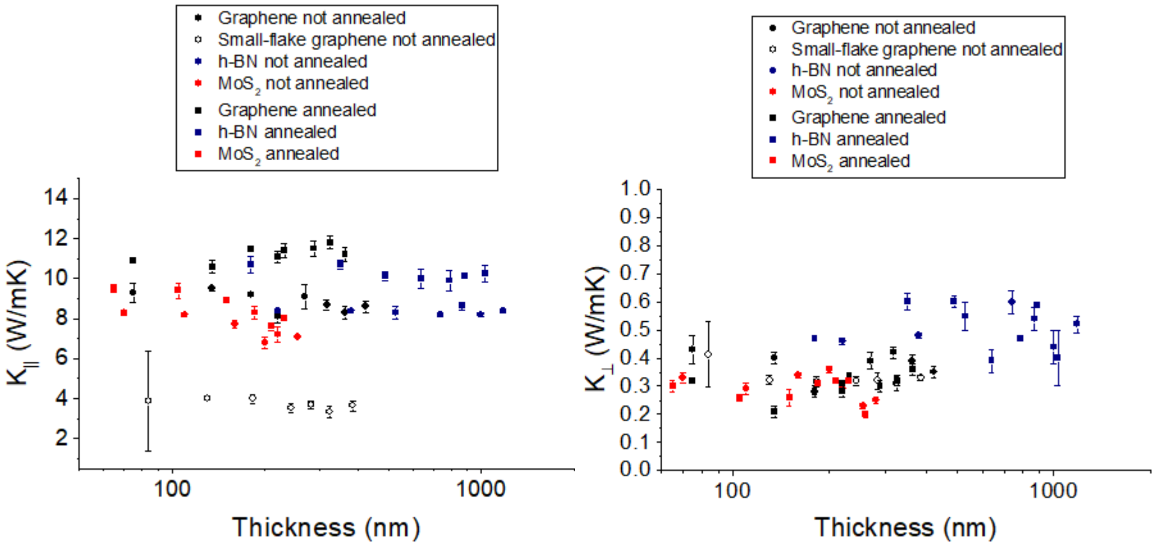

Figure S15. Extracted values for $K_\perp$ and $K_\parallel$ for all samples measured: graphene, small-flake graphene, h-BN and MoS_2, including data before and after annealing. Note that each data point is the result of several measurements on the same sample. No dependence on film thickness was observed.

In order to determine whether both $K_\parallel$ and $K_\perp$ can simultaneously be obtained from the FDTR data, we conduct sensitivity analyses on the dependence of the measured thermal phase to the parameters of interest. The phase sensitivity has been defined as $S_p = d\theta/dp$, where θ is the thermal phase and p the parameter of interest. Figure S16a shows a sample sensitivity plot for a representative graphene film in this work. The y-axis shows the amount the measured thermal phase would change if the respective parameter were to vary by 10%. The figure shows a clear separation in sensitivity to $K_\perp$ (Kz-Film) and $K_\parallel$ (Kr-Film) at high frequencies. Therefore, the two parameters can justifiably be extracted simultaneously from the experimental data. The thermal phase sensitivity is well above 0.1 deg per 10% change. This can be compared to the phase noise in the experiment, which is within 0.1 deg. Therefore, our experiment has sufficient sensitivity to determine the anisotropic thermal conductivity values for the 2D crystal films. Figure S16a also shows the most relevant parameters to the model that need to be accurately determined a priori, such as spot size, and Al transducer and SiO_2 thickness and conductivity. How uncertainties in these values affect our analysis is described below through Monte-Carlo analysis.

The ability to independently fit these parameters is further supported by the contour plots in Figure S16b, showing how $K_\perp$ and $K_\parallel$ are correlated in the model in a typical measurement. The dot in the center is the best fit value, and the blue (red) contour shows the combination of fitted values that yield 50% (100%) increase of the fit mean square error. These contours represent the 68% and 95% confidence intervals, respectively. Although the contours stretch diagonally, indicating some degree of correlation among the parameters, the range within which this occurs within reasonable error is small.

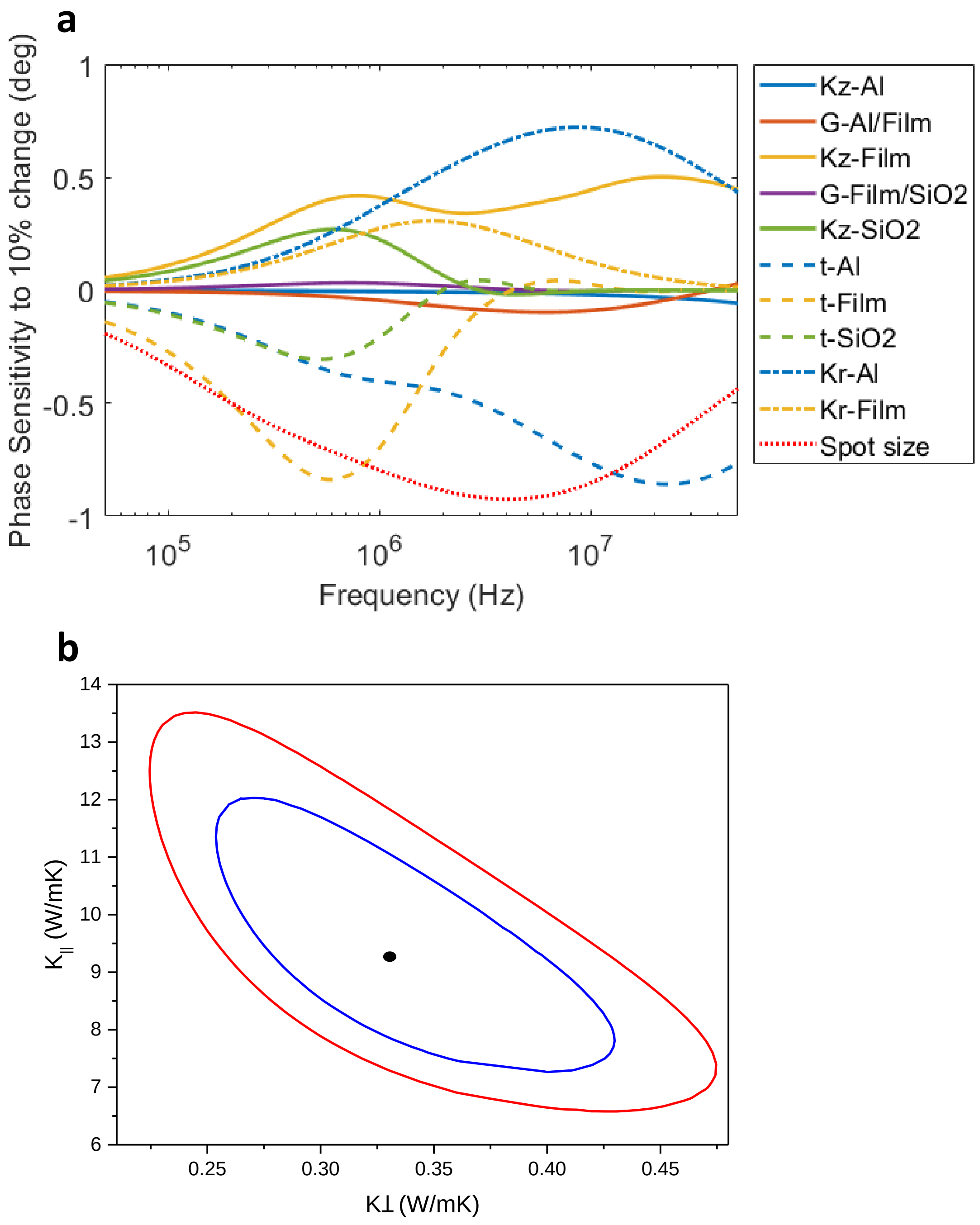

Figure S16. a Phase sensitivity of a representative 200 nm inkjet-printed graphene film having values for $K_{\perp}$ (Kz-Film) and $K_{\parallel}$ (Kr-Film) of 0.33 and 9.5 W/mK, respectively. Phase sensitivities to other relevant parameters in the model that are otherwise kept constant are also shown. **b** Contour of the correlation between $K_{\perp}$ and $K_{\parallel}$ as mean square error from best fit (single point) is increased by 50% (blue) and 100% (red), see text.

The errors in the obtained values for $K_\perp$ and $K_\parallel$ can be due to other assumptions in the thermal model. Estimates of error propagation of assumed parameters were done thorough Monte Carlo analysis, as we described previously [S6]. Briefly, in each iteration the thermal model parameters that were considered as known were drawn randomly from a normal distribution with a given expected value and estimated uncertainty distribution. This perturbed model was then fitted to the data to determine the unknown thermal conductivity values of the film. Repeating this procedure yields the uncertainty distribution in thermal conductivity values due to the combined uncertainties of all assumed parameters. The standard deviation for the assumed parameters was estimated from several independent measurements. The uncertainty in Al thickness was 4%, for the thermal conductivity of Al it was 5%, for the thickness of the SiO_2 it was 5%, for the thickness of the ink we used 4%, and for optical pump and probe spot size it was 4%. We also included the uncertainty in the thermal phase measurement based on the experimental noise of the FDTR signal, taken to be 0.1 degrees. To include uncertainty in C of the films, we randomly selected a value ranging from 85% to 100% of the bulk value. This is based on the fact that C can be lower than bulk due to porosity, but not greater. We performed 2,000 Monte Carlo iterations recording best fit thermal conductivity parameters and associated goodness of fit values, until the resulting distribution was adequately sampled. Example histograms are shown in Figure S17. Finally, the mean and standard deviation in thermal conductivity value was extracted from the Monte Carlo data by weighing each sample value by its associated goodness of fit, for statistical significance. The effect is that a large deviation from a known parameter value that yields a poor fit of the thermal model to the experimental data is weighted less in the process of obtaining the uncertainty in thermal conductivity.

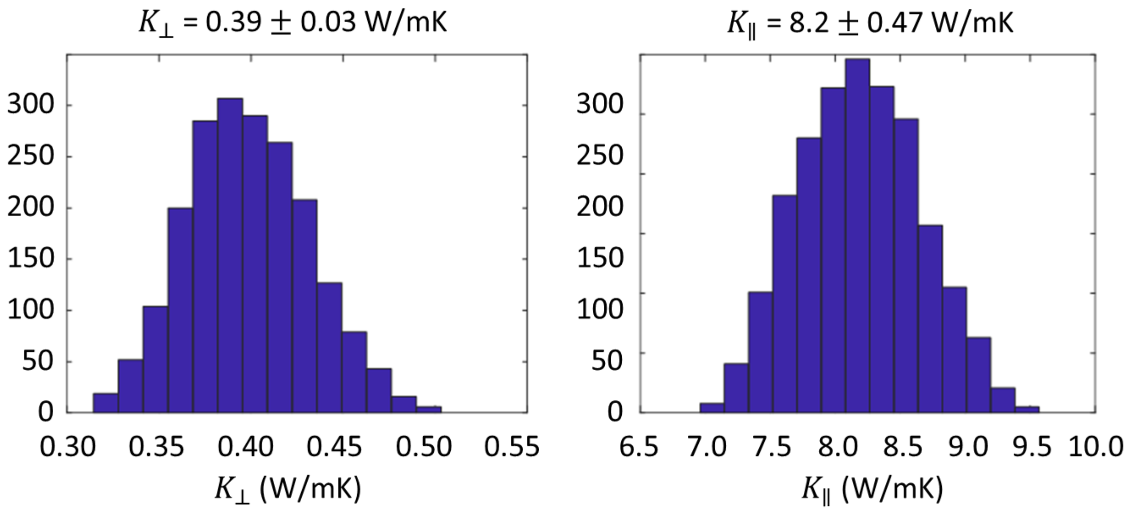

Figure S17. Monte Carlo histograms for uncertainty estimation for a 424 nm graphene film.

4. Additional details on density functional theory calculations

Thermal transport conductivities were calculated with the approach developed in [S14], by using phonon dispersions and anharmonic three-phonon scattering coefficients computed with density functional theory (DFT) within the plane-waves and pseudopotential approaches of the quantum-espresso package [S15-S17]. DFT calculations were done in the local-density (LDA) approximation [S118] using norm conserving pseudopotentials. According to literature [S16,S19-S21], simple LDA provides a very good description of vibrational properties for the three systems studied (possibly because of a cancellation of errors), in spite of the absence of the van der Waals correction terms usually necessary for lamellar materials.

For graphite we used the same computational details as those of [S16] obtaining the same phonon dispersions. In particular, we use the equilibrium lattice parameter *a*=2.44 Å and *c/a* is phenomenologically set to 2.664 to reproduce the acoustic branches phonon dispersion along the out-of-plane direction, see discussion in [S16]. For h-BN (AA' stacking) we used plane-waves cutoff of 110 Ry and an electronic integration grid of 12x12x8 k-points. The resulting equilibrium structure, *a*=2.478 and *c*=6.425 Å, is not different from that reported in [S19]. For MoS_2 (2H structure) the cutoff is 90 Ry and the electronic grid 10x10x3. The resulting equilibrium structure, *a*=3.140 Å and *c*=12.029 Å, is not substantially different from that reported in [S20,S21].

Following the standard approach [S15], phonon dispersions are obtained by Fourier interpolation from the dynamical matrices calculated exactly (within DFT) on a phonon wave-vector grid (8x8x6 and 6x6x4 for h-BN and MoS_2, respectively), by including long-range interaction though Born effective charges and dielectric constants as determined from DFT. The resulting dispersions reproduce well the measurements, Figure S18, and are in reasonable agreement with those already published [S19,S20].

Thermal conductivities are calculated with the approach described in [S14]. The integration of the Boltzmann-like equation is done on a grid of 45x45x9 phonon wavevectors for all the systems. The phonon-scattering energy-conservation delta-distribution is approximated with a Lorentzian with 10 cm^{-1} HWHM width (this relevant parameter is not reported in previous analogous calculations). Following [S16], the required anharmonic 3-phonon scattering coefficients are determined by Fourier interpolation of coefficients calculated on triplets of phonon wavevectors running on a grid. For graphite the grid is the same as that of [S22], while for h-BN and MoS_2 we used 2x2x2 grids testing up to 4x4x2. Note that for MoS_2, the use of these coarse grids should provide an intrinsic conductivity less precise than the one reported in [S21]. This problem, however, mildly affects the conductivities reported in Figure 3 in the main text, which are shown in a region (small *L*) where the conductivity is mostly determined by the harmonic properties of the system (i.e. not by the three-phonon scattering). The resulting intrinsic conductivities at room temperature and natural isotope composition in-plane (out-of-plane) are 623 (5.8), 540 (4.2), and 160 (7.2) W/mK for graphite, h-BN, and MoS_2, respectively.

These are the values that one would obtain by letting L go to infinity in Figure 3 of the main article.

Finally, as a reference, Figure S19 reports the calculated “dirty” conductance G^* as a function of the cutoff energy E_c. Both G^* and E_c are defined in the main text. The figure also reports the conductance spectral decomposition defined as $g(E) = \frac{1}{2}\langle\frac{dn}{dT}\epsilon v\delta(\epsilon - E)\mathcal{T}\rangle$, where δ is the Dirac delta function.

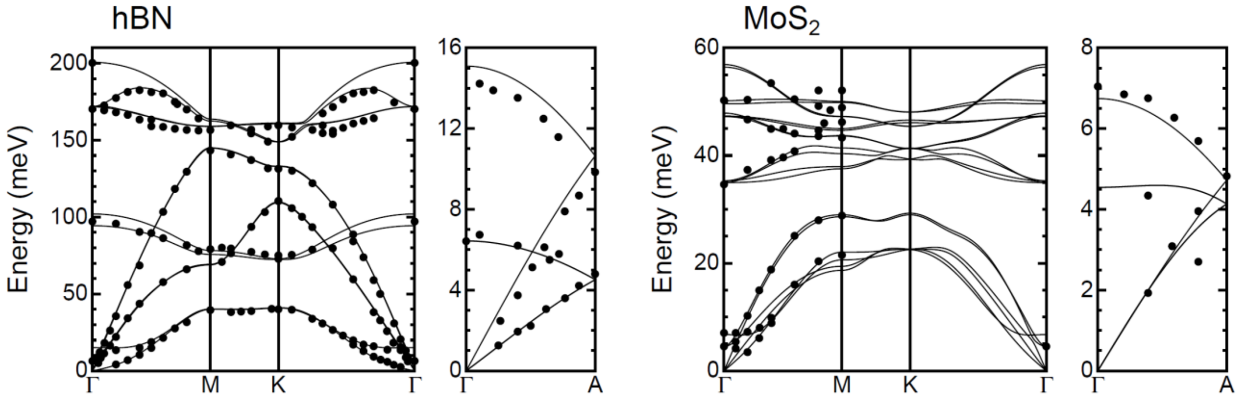

Figure S18. Calculated phonon dispersions along the high symmetry lines of the *hcp* structure for h-BN (AA' stacking) and the 2H-MoS_2 structure. Dots are measurements scanned from [S23-S25].

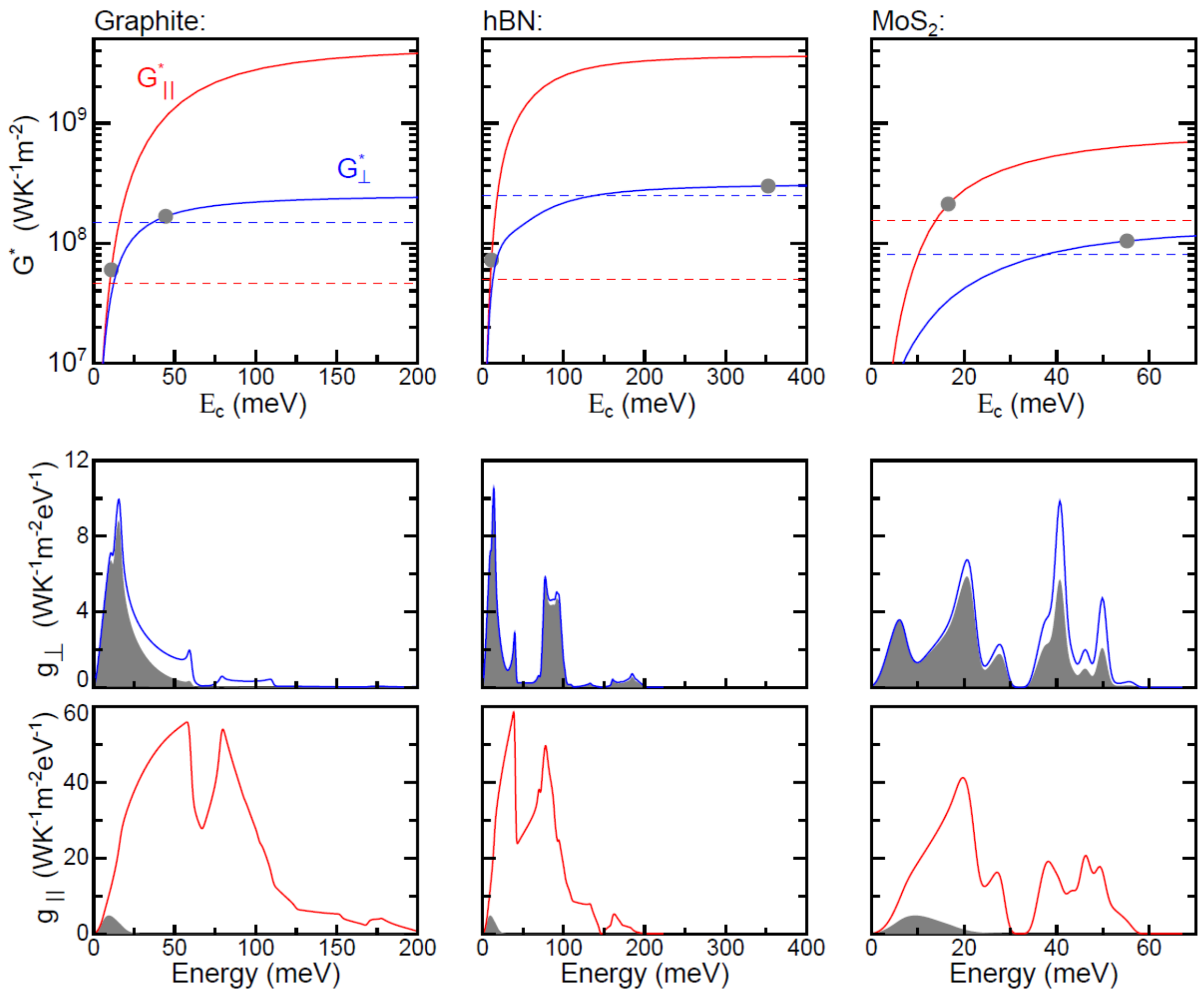

Figure S19. Upper panels: Dirty-interface thermal conductance (G^*) calculated as a function of the cut-off energy (E_c) for the three materials. ∥ (⊥) refers to the in-plane (out-of-plane) transport direction. The grey dots correspond to the E_c values chosen to fit measurements. The horizontal dashed lines are the measured conductivities divided by the average flake dimension along the transport direction and are shown only as references. Middle and bottom panels: calculated conductance spectral decomposition (defined in the text). Blue and red lines are for the "ideal" conductance ($\mathcal{T} = 1$, $E_c = +\infty$). The profile of the grey areas correspond to the "dirty" conductance associated to the E_c of the dots in the upper panels.

References

[S1] D. McManus, S. Vranic, F. Withers, V. Sanchez-Romaguera, M. Macucci, H. Yang, R. Sorrentino, K. Parvez, S.-K. Son, G. Iannoccone, K. Kostarelos, G. Fiori, C. Casiraghi, Water-based and biocompatible 2D crystal inks for all-inkjet-printed heterostructures, *Nature Nanotechnology* **12**, 343 (2017).
[S2] Y. Hernandez, V. Nicolosi, M. Lotya, F.M. Blighe, Z. Sun, S. De, I.T. McGovern, B. Holland, M. Byrne, Y.K. Gun'Ko, *et al.* High-yield production of graphene by liquid-phase exfoliation of graphite, *Nature Nanotechnology* **3**, 563 (2008).
[S3] J.N. Coleman, M. Lotya, A. O'Neill, S.D. Bergin, P.J. King, U. Khan, K. Young, A. Gaucher, S. De, R.J. Smith, *et al.* Two-dimensional nanosheets produced by liquid exfoliation of layered materials, *Science* **331**, 568 (2011).
[S4] H. Yang, F. Withers, E. Gebremedhn, E. Lewis, L. Britnell, A. Felten, V. Palermo, S. Haigh, D. Belijonne, C. Casiraghi, Dielectric nanosheets made by liquid-phase exfoliation in water and their use in graphene-based electronics, 2D Materials **1**, 011012 (2014).
[S5] M. Rahman, M. Shahzadeh, P. Braeuninger-Weimer, S. Hofmann, O. Hellwig, S. Pisana, Measuring the thermal properties of anisotropic materials using beam-offset frequency domain thermoreflectance, *Journal of Applied Physics* **123**, 245110 (2018).
[S6] M. Rahman, M. Shahzadeh, S. Pisana, Simultaneous measurement of anisotropic thermal conductivity and thermal boundary conductance of 2-dimensional materials, *Journal of Applied Physics* **126**, 205103 (2019).
[S7] Y. Takahashi, T. Azumi, Y. Sekine, Heat capacity of aluminum from 80 to 800 K, *Themochimica Acta* **139**, 133 (1989).
[S8] A.T.D. Butland, R.J. Maddison, The specific heat of graphite: an evaluation of measurements, *Journal of Nuclear Materials* **49**, 45 (1973).
[S9] A.S. Dworkin, D.J. Sasmor, E.R. Van Artsdalen, The thermodynamics of boron nitride: low-temperature heat capacity and entropy; heats of combustion and formation, *Journal of Chemical Physics* **22**, 837 (1954).
[S10] L.S. Volovik, V.V. Fesenko, A.S. Bolgar, S.V. Drozdova, L.A. Klochkov, V.F. Primachenko, Enthalpy and heat capacity of molybdenum disulfide, *Soviet Powder Metallurgy and Metal Ceramics* **17**, 697 (1978).
[S11] C.J. Foss, Z. Aksamija, Quantifying thermal boundary conductance of 2D-3D interfaces, *2D Materials* **6**, 025019 (2019).
[S12] A.J. Gabourie, C. Koroglu, E. Pop, Substrate-dependence of monolayer MoS2 thermal conductivity and thermal boundary conductance, *arXiv preprint*, arXiv:2204:11381 (2022).
[S13] D. R. Lide, *CRC Handbook of Chemistry and Physics*, Taylor & Francis (2007).
[S14] G. Fugallo, M. Lazzeri, L. Paulatto, F. Mauri, Ab initio variational solution for lattice thermal conductivity in diamond, *Physical Review B* **88**, 045430 (2013).
[S5] S. Baroni, S. de Gironcoli, S. Dal Corso, P. Giannozzi, Phonons and related crystal properties from density-functional perturbation theory, *Review of Modern Physics* **73**, 515 (2001).
[S16] L. Paulatto, F. Mauri, M. Lazzeri, Anharmonic properties from a generalized third-order ab initio approach: theory and applications to graphite and graphene, *Phyisical Review B* **87**, 214304 (2013).
[S17] P. Giannozzi, S. Baroni, N. Bonini, M. Calandra, R. Car, C. Cavazzoni, D. Ceresoli, G.L. Chiarotti, M. Cococcioni, I. Dabo, A. Dal Corso, S. de Gironcoli, S. Fabris, G. Fratesi, R. Gebauer,

U. Gerstmann, C. Gougossis, A. Kokalj, M. Lazzeri, L. Martin-Samos, N. Marzari, F. Mauri, R. Mazzarello, S. Paolini, A. Pasquarello, L. Paulatto, C. Sbraccia, S. Scandolo, G. Sclauzero, A.P. Seitsonen, A. Smogunov, P. Umari, R.M. Wentzcovitch, Quantum ESPRESSO: a modular and open-source software project for quantum simulations of materials, *Journal of Physics: Condensed Matter* **21**, 395502 (2009).
[S18] J.P. Perdew, A. Zunger, Self-interaction correction to density-functional approximations for many-electron systems, *Physical Review B* **23**, 5048 (1981).
[S19] P. Jiang, X. Qian, R. Yang, L. Lindsay, Anisotropic thermal transport in bulk hexagonal boron nitride, *Physical Review Materials* **2**, 064005 (2018).
[S20] A. Sood, F. Xiong, S. Chen, R. Cheaito, F. Lian, M. Asheghi, Y. Cui, D. Donadio, K. E. Goodson, E. Pop, Quasi-ballistic thermal transport across MoS2 thin films, *Nano Letters* **19** 2434 (2019).
[S21] S. Chen, A. Sood, E. Pop, K.E. Goodson, D. Donadio, Strongly tunable anisotropic thermal transport in MoS2 by strain and lithium intercalations: first-principles calculations, *2D Materials* **6**, 025033 (2019).
[S22] G. Fugallo, A. Ceppellotti, L. Paulatto, M. Lazzeri, N. Marzari, F. Mauri, Thermal conductivity of graphene and graphite: collective excitations and mean free paths, *Nano Letters* **14**, 6109 (2014).
[S23] J. Serrano, A. Bosak, R. Arenal, M. Krisch, K. Watanabe, T. Taniguchi, H. Kanda, A. Rubio, L. Wirtz, Vibrational properties of hexagonal boron nitride: inelastic x-ray scattering and ab initio calculations, *Physical Review Letters* **98**, 095503 (2007).
[S24] R.J. Nemanich, S.A. Solin, R.M. Martin, Light scattering study of boron nitride microcrystals, *Physical Review B* **23**, 6348 (1981).
[S25] N. Wakabayashi, H.G. Smith, R.M. Nicklow, Lattice dynamics of hexagonal MoS2 studied by neutron scattering, *Physical Review B* **12**, 659 (1975).
